# Impact of instrumental settings in electrospray ionization ion trap mass spectrometry on the analysis of *O*-methoxyethyl-*O*-methyl cellulose: a comprehensive quantitative evaluation

**DOI:** 10.1007/s00216-022-04095-3

**Published:** 2022-05-03

**Authors:** Sarah Schleicher, Dominik Horoba, Philip Krafzig, Petra Mischnick

**Affiliations:** Institute of Food Chemistry, Technische Universität Braunschweig, Schleinitzstr 20, 38106 Braunschweig, Germany

**Keywords:** Oligosaccharide ethers, Electrospray ionization ion trap mass spectrometry, Quantitative mass spectrometry, Substituent distribution, Hydroxyethyl(methyl)cellulose

## Abstract

The hydroxyethyl substitution along and among the polymer chains of respective cellulose ethers (HEC and HEMC) can be analyzed by ESI-IT-MS after permethylation of the free OH-groups, partial hydrolysis, and *m*ABA labeling. This method requires the correct quantification of the molar ratios of the constituents belonging to a particular degree of polymerization (DP) with respect to their numbers of MeOEt and Me groups without any discrimination along the MS analysis pathway. The influence of the chemistry on the ionization and the impact of the voltages controlling the ion transport (Cap Exit, Octopoles) and the ion storage efficiency (Trap Drive, TD) on a relative quantification were studied using binary equimolar mixtures of cellobiose with increasing number of methoxyethyl and decreasing number of methyl groups (Δ *m/z* 88, 2× MeOEt). No suppression effects were observed in concentration-dependent measurements. Choice of Cap Exit is especially crucial for low *m/z* with less MeOEt residues. An equation describing the relationship between Oct 2 DC, *m/z*, and TD_max_ (TD at maximum intensity) was established from the experimental data and applied to calculate TD_max_ for higher DPs (larger COS). Optimized conditions allowed to determine the correct molar ratio of binary mixtures. Measurements of overlapping *m/z* segments and subsequent interrelation of the data gave complete substitution profiles for MeOEt/Me celluloses in accordance with reference data. The study generally makes aware of potential erroneous quantification in ESI-IT-MS analysis using internal standards of similar chemistry or in relative quantification of analytes, even for those with related structures.

**Graphical abstract:**

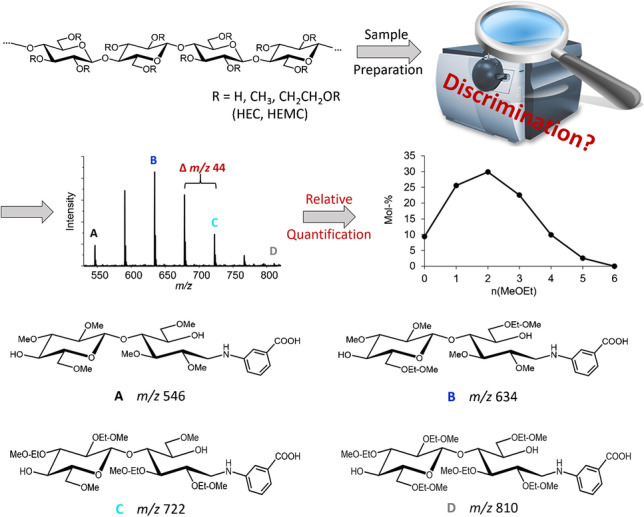

**Supplementary Information:**

The online version contains supplementary material available at 10.1007/s00216-022-04095-3.

## Introduction

Cellulose is chemically modified in various ways to generate bio-based materials with a wide range of properties, especially to overcome insolubility of cellulose. While cellulose esters are used as such due to their thermoplastic properties, cellulose ethers play a major role as additives in order to control, for instance, viscosity and rheology, adhesive properties, and gelling. Beside the anionic carboxymethyl cellulose (CMC), methyl cellulose (MC), hydroxyethyl- and hydroxypropylcellulose (HEC, HPC), and mixed ethers as HEMC and HPMC are the most important ones. The main fields of application are the construction area, food, cosmetics, and pharma [1]. Beside the molecular weight distribution, the exact chemical composition of such complex materials has a crucial impact on the physicochemical properties. Beside the average degree of substitution (DS) or in case of hydroxyalkyl ethers, additionally, the average molar degree of substitution (MS); the distribution on the positions 2, 3, and 6 of the glucosyl unit; and the pattern along and among the cellulose chains as well are of high interest [2]. Reaction conditions together with the mechanistic question of kinetic or thermodynamic control of the respective reaction have a critical impact on the regioselectivity and the homogeneity of the reaction over the entire material [2]. The substituent pattern in the glucosyl unit, expressed as the molar portions of the eight different un-, mono-, di-, and tri-*O*-substituted constituents, can be determined after total depolymerization by various separation and detection methods. Gas liquid chromatography (GLC) is most frequently applied due to its high separation efficiency and its well established coupling with MS. The MS spectra of the corresponding alditol acetates allow to deduce the positions of substitution. Furthermore, GLC-FID in combination with the effective carbon response (ECR) concept enables the quantification of the molar ratios of the constituents without calibration requiring standard compounds [3–5]. In the case of hydroxyalkyl ethers, the occurrence of tandem substitution and consequently increasing possibilities of substitution patterns makes GLC-MS superior to all alternatives [5–8].

More challenging than the monad analysis of cellulose ethers is the analysis of the substituent distribution in the second and third dimension, i.e., along and among the polymer chains [2, 3]. Information on pattern probabilities is available by MS analysis of the substituent distribution in oligosaccharides (diads, triads etc.) obtained by partial random hydrolysis. The molar portions of all groups of constitutional isomers, belonging to the cello-oligosaccharides (COS) of a particular degree of polymerization (DP), are quantified. The profiles obtained for each individual DP are compared with a random distribution calculated from the independently determined molar portions of *n*-fold substituted glucosyl units [9–13]. The deviation of the experimental from the theoretical distributions indicates the type and measure of heterogeneity [2, 4, 9, 13–15]. For an exact relative quantification by electrospray ionization ion trap-mass spectrometry (ESI-IT-MS), it must be ensured that no discrimination occurs, nor during ionization due to the chemistry and matrix effects [16, 17], neither during ion transportation and mass analysis due to varying *m/z* of the analytes. The only available control parameter with regard to the plausibility of the results is the average DS or MS, respectively, calculated from the evaluated MS data. It should be constant for each DP and in agreement with the average DS (or MS) of the entire material. Any significant deviation from the average DS (MS) or any increasing or decreasing trend of DS (MS) with DP is a hint to bias and thus erroneous results. While in case of MC, in order to overcome potential sources of discrimination, chemical uniformity and a narrow *m/z* range can be achieved by permethylation with MeI-*d*_*3*_ (internal isotope labeling) [14, 15, 18, 19]; such leveling of differences in chemistry and mass is not possible for hydroxyalkyl derivatives. Each reaction performed at the glucosyl OH groups will also take place at the OH functions of the substituent and thus maintain the mass difference. These types of ethers have been permethylated in order to level the methyl pattern in HEMC (or HPMC, respectively) and to reduce differences in polarity (capping of all OH). Furthermore, the chemical difference in sodium complexation, the typical way of positive ion formation of carbohydrates, was overcome by labeling the oligosaccharides at their reducing end.

By the introduction of the more flexible, crown-ethers resembling *O*-(CH_2_CH_2_-*O*)_n_-CH_3_ segments at the carbohydrate backbone, sodium complexation properties are significantly enhanced. To generate charge-controlled positive ions, propyl amine has been introduced by reductive amination and subsequently quaternized [7, 8, 10]. By MALDI-ToF-MS, the average hydroxyalkyl MS was constantly met for all DPs evaluated for HEMCs and HPMCs with a maximum MS of 0.17 (HE) [7] and 0.21 (HP) [8], respectively. Measuring the same samples by ESI-IT-MS showed a decreasing MS with DP. Alternatively, aminobenzoic acid (ABA) was introduced for negative ion formation [11, 12]. By this modification and additional adaption of measurement conditions, the trend of decreasing MS with DP could be partly leveled. Beside the introduction of a defined charge, labeling also makes the analytes chemically more similar. But nevertheless, the mass differences, 44 Da per HE and 58 Da per HP, remain. Already for the ABA derivatives of the smallest oligosaccharide, DP2, the mass range of interest spans from *m/z* 546 (*n*(HE) = 0) to 810 (*n*(HE) = 6). Therefore, a deeper understanding of measurement parameters in ESI-IT-MS, i.e., how they affect the quantitative analysis of HE(M)C-derived COS derivatives, is necessary. It should be emphasized that this type of analysis does not aim for absolute concentration data, but the determination of the relative molar ratios of all constituents belonging to the COS of a particular DP [9].

**Fig. 1:**
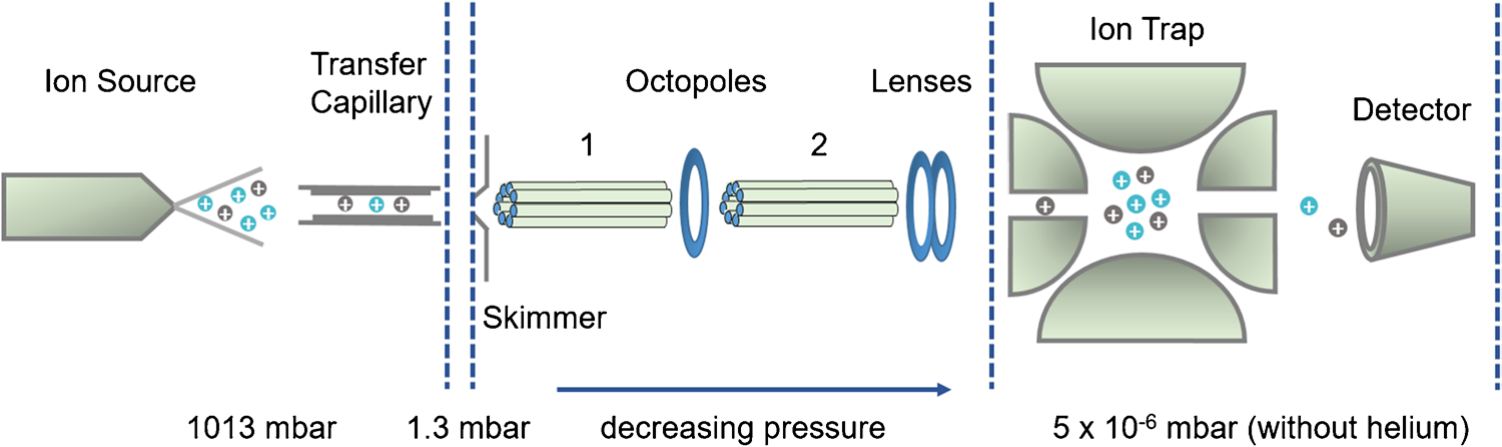
Instrumental set-up of the ESI-IT-MS used in this study (prepared according to the information for the HCT Ultra ETD II, Bruker Daltonics GmbH & Co. KG, Bremen, Germany) [19]

We recently reported on a comprehensive quantitative study about the impact of instrumental settings in ESI-IT-MS on the accuracy and precision of the determination of the molar ratios of isotopologous Me/Me-*d*_*3*_ COS [19]. In addition to potential sources of bias during the ESI process, the instrumental settings were studied which can cause discrimination of ions according to their *m/z* during ion transportation from the ion source to the mass analyzer and with regard to storage in the ion trap and finally detection. Kruve et al. reported on such optimization for pesticide analysis [20]. The ESI-IT-MS used in this study (HCT Ultra ETD II, Bruker) is schematically shown in Fig. 1.

An ESI-IT-MS device can be divided into four areas: the ionization unit, the transportation unit, consisting of the transfer capillary, the skimmer as well as the octopoles and lenses, and finally the ion trap followed by the detector. In the case of the Bruker HCT Ultra ETD II the nebulizer needle is grounded. The instrument can be operated in two different modes. The so-called *smart mode* allows convenient operation of the device by the user. Voltages of the transportation and focusing unit and of the mass analyzer are indirectly controlled.

On the contrary, in the so-called *expert mode*, the settings of the transportation unit and the ion trap can be adjusted directly and largely independently. These are the voltages of the skimmer, the capillary exit (Cap Exit*)*, the DC and RF voltages of the octopoles (Oct 1 and Oct 2 DC; Oct RF), the voltage of the lenses, and the RF voltage applied to the ring electrode of the ion trap (*Trap Drive*, TD). The voltage of the end plate is fixed at −500 V. The role of the individual voltages for a similar quadrupole ion trap ESI-MS (Agilent) has been reported by Kruve et al. [20] and has been discussed in more detail in our preceding paper on the relative quantification of Me/Me-*d*_*3*_ COS isotopologs [19].

The highest impact on the ion intensity is related to TD (amplitude of the RF voltage of the ion trap), Oct 2 DC and Oct RF as well as Cap Exit. Therefore, in our studies dealing with potential discrimination effects during a relative quantification of MeOEt/Me-COS analytes, we focused on these settings.

**Fig. 2:**
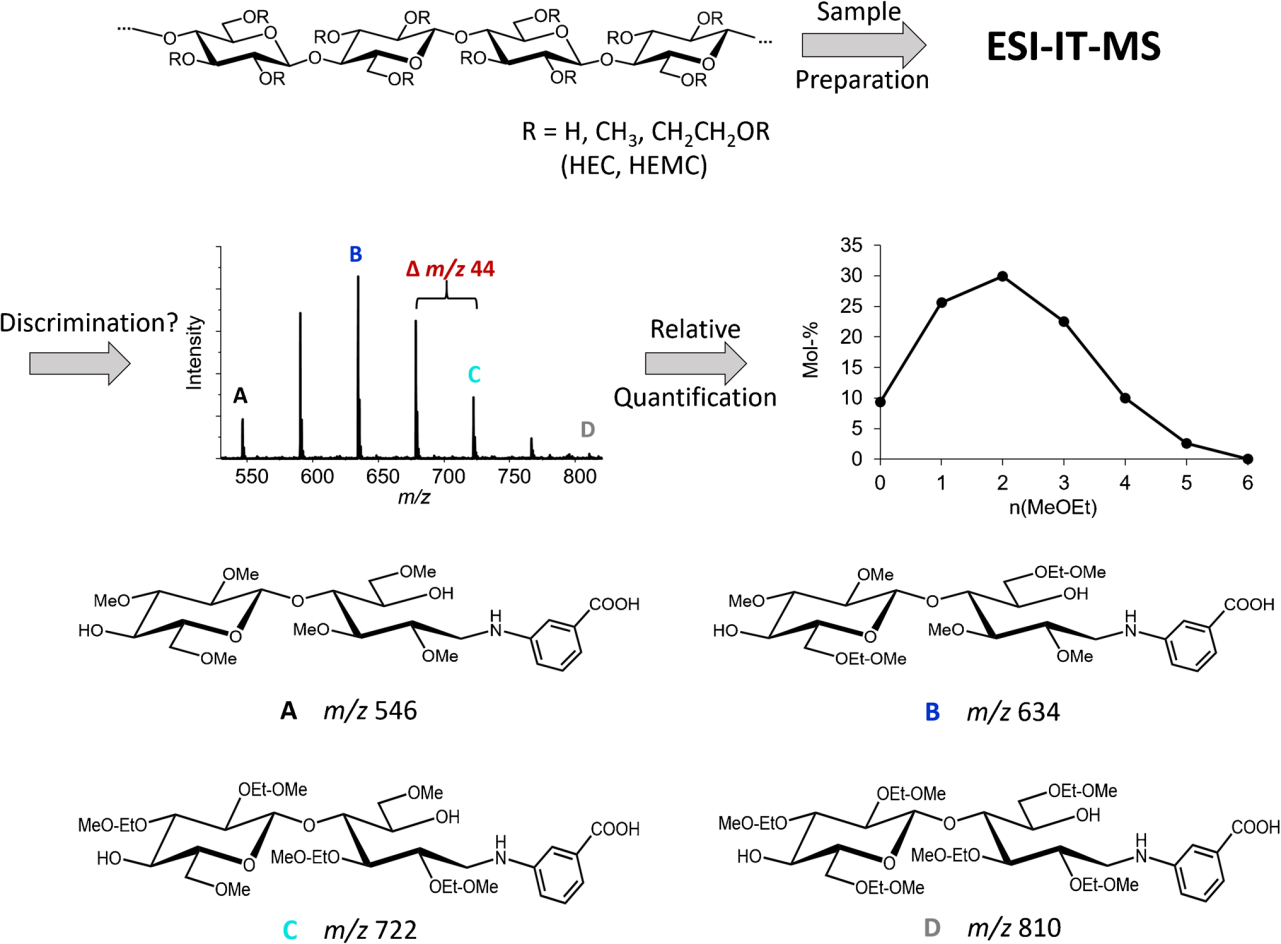
Scheme of the determination of the substituent distribution of cellulose ethers along and over the polymer chains by ESI-IT-MS; structures of selected cellobiose derivatives used as model compounds in this study aimed to exact quantification of their molar ratios are shown

While in the study of Me/Me-*d*_*3*_ COS isotopologs the two confining permethylated and perdeuteromethylated COS of DP2 to 6 were chosen as model compounds, in case of the MeOEt/Me COS, we decided to divide the *m/z* range of interest in smaller segments, since the entire mass range of a particular DP is very wide. Furthermore, we used *m*ABA labeled derivatives, because of already observed differences in ionization efficiency in case of sodium complexation. Therefore, in order to study the accuracy and precision of the quantitative ESI-IT-MS analysis of MeOEt/Me COS, we prepared equimolar binary mixtures of *m*ABA labeled cellobiose. These consisted of two glucosyl units with *n* Me and *m* MeOEt groups in both glucosyl units, starting with *n/m* = 3/0 (A) and 2/1 (B), i.e., consisting of two 2,3,6-tri-*O*-methylated glucosyl units and of two 2,3-di-*O*-methyl-6-*O*-methoxyethylated ones, respectively (mixture AB), continuing with *n/m* = 2/1 and 1/2 (C) (mixture BC), and finally *n/m* = 1/2 and 0/3 (D) (mixture CD). These model compounds were obtained from corresponding regioselectively substituted cellulose ethers by partial hydrolysis, high-performance liquid chromatography (HPLC) fractionation, and reductive amination with *m*ABA. In Fig. 2, the scheme of this cellulose ether analysis and the structures of the studied model compounds are presented.

The absolute concentration and exact molar ratio (MR) in the binary mixtures were determined by HPLC/UV. First, the influence of the RF voltage in the IT (TD) on the intensity ratio of detected ions (IR) was investigated systematically. For comparability, the measured IR were corrected by MR to normalize all results to equimolarity. Afterwards the voltages affecting the ion transportation were varied. Finally, the impact of Cap Exit on ion ratios was also studied. If there is any bias in the ESI process, this should become visible above the saturation point (commonly at about 10^-5^ M). Therefore, total concentrations of the binary mixtures were varied from 1 ∙ 10^−9^ M up to 1 ∙ 10^−4^ M over six orders of magnitude. Finally, it was checked whether the observed effects play a role in the analysis of permethoxyethylated MC or a permethylated HEMC (real sample).

## Materials and methods

### Materials

Except DMSO (≥ 99.5 % for synthesis) and trifluoroacetic acid (≥ 99.9 %), purchased from Roth, all other chemicals were from Sigma-Aldrich/Merck with the following purity: iodomethane (MeI) (≥ 99 %), 2-bromoethyl methyl ether (MeOEtBr) (≥ 85 %), 2-picoline borane (≥ 95 %), *m*-aminobenzoic acid (*m*ABA) (≥ 99 %), FeCl_3_ (97 %), sodium hydroxide pellets (≥ 97 %), acetic acid (HOAc) (≥ 99.8 %), and methyllithium solution (1.6 M in diethyl ether).

Acetonitrile (ACN) and methanol (MeOH) were purchased from Riedel-de Haen, toluene, and dichlormethane (DCM) from Fisher Chemicals. For ESI-MS measurements, LC-MS grade was used, for chromatographic fractionation and all other applications HPLC-grade.

As starting material for the synthesis of the model compounds cellulose acetate (40 % acetyl groups) from Fluka (A, D) and 6-*O*-tritylcelluose (B, C), a gift from Prof. Th. Heinze, University of Jena, was used, respectively. The HEMC (MS_HE_ 0.35, Zeisel) and MC1 (DS_Me_ 1.29) and MC2 (DS_Me_ 1.95) were from former DOW Wolff Cellulosics GmbH, Bomlitz, Germany.

### Instrumentation

MS studies were performed on a HCT Ultra ETD II (Bruker Daltonics, Bremen, Germany), equipped with an ion trap (IT) and a conversion detector with a photomultiplier (Daly detector). For direct infusion experiments, the general measurement parameters were as follows: nitrogen was used as dry gas (6 L min^−1^, 300 °C) and nebulizer gas (10 psi), capillary +3.5 kV, end plate offset -500 V, skimmer -40 V, Oct 1 DC -8 V, lens 1 +5 V, lens 2 +60 V, and ion charge control 70,000 as well as maximum accumulation time 200 ms. Further parameters are specified in the text (Cap Exit, Oct 2 DC, Oct RF, TD).

For LC, the ESI-MS was coupled to an Agilent LC system equipped with a binary pump (1100 series), autosampler (1200 series), and a DAD detector (1100 series). The following parameters were changed compared to direct infusion: dry gas (10 L min^−1^, 365 °C) and the nebulizer gas (50 psi) as well as the capillary voltage (+4.5 kV). The RF voltage of the ion trap, the DC voltage Oct 2, and the RF voltage of the octopoles was set indirectly using the smart parameter mode (TM, mass of the analyte; compound stability, 1000 %; Trap Drive Level, 100 %).

### Preparation of 2,3,6-*O*-methyl (A), 6-*O*-methoxyethyl-2,3-*O*-methyl (B), 2,3-*O*-methoxyethyl-6-*O*-methyl (C), and 2,3,6-*O*-methoxyethyl (D) cellobioses

The unlabeled cellobiose derivatives for standards A, B, C, and D (see Fig. 2) were obtained by semi-preparative HPLC (*see* *Fractionatio**n*) of the corresponding cellulose derivatives after partial hydrolysis (see *Partial hydrolysis*).

Precursors of model compound A (fully *O*-methylated) and D (fully *O*-methoxyethylated) were synthetized from cellulose acetate (1 g) according to the alkylation method of Ciucanu and Kerek (NaOH as base) [21] and the Hakomori Method (Li-Dimsyl as base) [22] as described in [19]. As alkylation reagent MeI and MeOEtBr was used, respectively.

Starting material for the synthesis of 2,3-*O*-methyl-6-*O*-methoxyethyl (➔B) and 2,3-*O*-methoxyethy-6-*O*-methyl cellulose derivatives (➔C) was 6-*O*-tritylcellulose (each 0.7 g). In the first step, OH groups at position 2 and 3 were peralkylated with MeI (B-COS) or with MeOEtBr (C-COS), respectively [19]. Subsequently, the protecting group at position 6 was removed by FeCl_3_ (3 eq per AGU) in DCM (14 μmol mL^−1^) for 3 h at room temperature (r.t.). Samples were purified by dialysis against acetone until colorlessness, then against water and freeze dried. Completeness of deprotection was checked by the absence of the aromatic C=C absorption at 1444 and 1490 cm^−1^ as well as of the aromatic C-H at 3060 cm^−1^ by ATR-IR spectroscopy (s. ESM section A, Fig. S1). Subsequently, position 6 was alkylated with MeOEtBr (B-COS) or MeI (C-COS), respectively.

#### Partial hydrolysis

The cellulose ethers, about 0.3–1.0 g, were partially hydrolyzed. First, the material was swollen overnight in water (compound B and C), or acetone/H_2_O (50/50, v/v) (compound A and D), respectively, at a concentration of 2.4 mg mL^−1^. Concentrated TFA was added (final concentration 2 M), and the mixture was kept at 120 °C for 22 (A and D) or 45 min (B and C), respectively. After cooling to r.t. acetone was evaporated, if applicable, and the hydrolysates were freeze dried. The samples were dissolved in ACN/H_2_O (30/70; v/v) to a total concentration of 60 mg mL^−1^.

#### Fractionation

Fractionation of the partially hydrolyzed cellulose ethers was performed on a semi-preparative HPLC system from Agilent (series Infinity 1260) coupled with an ELSD detector (Softa Model 300 S ELSD) (s. ESM section A, Fig. S2): column, RP-C_18_-column (Phenomenex, Gemini 5 μm, 250 × 10 mm); flow rate, 2 mL min^−1^; eluent, H_2_O + 1% HOAc (A) and ACN + 1% HOAc (B); t = 0, 80% A and 20% B; linear gradient to 0% A and 100% B in 50 min; and injection volume, 100 μL (6 mg COS per run). Since the COS are not UV-active, the retention time and the capacity of the column were checked using the ELSD detector. The fractionation was carried out without using a detector. The purity of the respective fractions of DP2 was checked by ESI-MS (direct infusion) prior to combining them. Afterwards, ACN was evaporated, and the remaining aqueous phase was freeze dried. The residues were dissolved in ACN and quantitatively transferred in a 4 mL vial. The solvent was removed in a stream of nitrogen. The residue was weighed and dissolved in 4 mL ACN/H_2_O (70/30; v/v).

### Standard solutions of binary mixtures of mABA-labeled A, B, C, and D of DP2 (A + B, B + C, C + D)

For the preparation of the binary mixtures of cellobiose derivatives A, B, C, and D, a defined volume of each COS standard, aiming to standard solutions of *c* = 4∙10^−4^ M, was labeled with *m*ABA (see *Labeling with* m*-aminobenzoic acid*). The concentration of each standard solution was determined by HPLC-UV-MS (*n* = 6) (see *HPLC-UV-MS*). Quantification was based on the UV signal. The MS data was used to check the purity of the peaks. In case of occurring regioisomers of the target compound, their contribution was evaluated according to the extracted ion chromatograms of these analytes with the same *m/z*, since they will not be differentiated in ESI-IT-MS with syringe pump infusion, later applied. The equimolar binary mixtures AB, BC, and CD were prepared by combining corresponding volumes of the standard solutions with a total concentration of 1 ∙ 10^−4^ M in ACN/H_2_O (90/10), i.e., 0.5 ∙ 10^−4^ M each. The actual total concentration as well as the molar ratios (MR) of B:A, C:B, and D:C were also determined by HPLC-UV-MS (*n* = 6).

#### Labeling with *m*-aminobenzoic acid

A defined volume of COS-standard solution (800 nmol analyte) was transferred to a 500 μL V-vial and evaporated under a stream of nitrogen at r.t. The residue was dissolved in 200 μL MeOH, and 120 μL *m*ABA-solution (*c* = 10 nmol μL^−1^ in MeOH) and 60 μL HOAc were added. The sample was heated at 40 °C for 30 min. After cooling to r.t., 20 μL of 2-picoline borane was added (*c* = 60 nmol μL^−1^ in MeOH), and the mixture was again heated to 40 °C for 45 min. Subsequently, the solvent was removed in a stream of nitrogen at r.t. The residue was dissolved in ACN. Success of labeling was checked by ESI-IT-MS in positive and negative mode. If the labeling process was complete, the sample was quantitatively transferred into a 2 mL flask with ACN/H_2_O (70/30; v/v) (c ~ 4 ∙ 10^−4^ M).

#### HPLC-UV-MS

The concentration of the solutions of *m*ABA-labeled standard and of the binary mixtures was determined by HPLC-UV-MS using the UV signal at 254 nm. Separation was performed on a RP-C_18_-column (Phenomenex Kinetex RP18, 100 × 2.1 mm, 2.6 μm) at 40 °C: flow rate, 0.2 mL min^−1^; eluent, H_2_O + 1% HOAc (A) and ACN + 1% HOAc (B); *t* = 0, 90% A and 10% B; linear gradient to 20% A and 80% B in 30 min; and injection volume, 3 μL (COS standards) and 8 μL (standard binary mixtures), *n* = 6. Quantification was based on an external calibration with *m*ABA-labeled 2,3,6-tri-*O*-methyl glucose standard (0.38–3.82 ∙ 10^−4^ M). For MS parameter, see chapter *Instrumentation*.

### MS measurements of binary mixtures of MeOEt_m_/Me_n_-cellobioses

The influence of the RF voltage of the IT (set by TD), the DC voltage of Oct 2, and the RF voltage of the octopoles as well as the voltage of the capillary exit on the relative ion intensities of the *m*ABA-labeled MeOEt_m_Me_n_-cellobioses in the binary mixtures AB, BC, and CD was studied systematically. The binary mixtures were applied with a concentration of 1∙10^−6^ M by syringe pump infusion at a flow rate of 200 μL h^−1^. Data were recorded in the negative mode. The general measurement parameters are mentioned under *Instrumentation*, whereas the particular measurement parameters of the individual experiments (variation of TD, Oct 2 DC, Oct RF, Cap Exit) are given under *Results and discussion*. For each measurement, 200 scans were accumulated (*n* = 3). To check discrimination effects during the ionization, a concentration series of the binary mixtures was measured (1 ∙ 10^−9^ M to 1 ∙ 10^−4^ M, *n* = 5).

MS data were evaluated using the Bruker Daltonics Data Analysis software. Peak intensities of the mass spectra were corrected for the corresponding isotope signals by adding their calculated intensities to the main peak after noise correction. The isotope distribution of the model compounds was calculated with the program *Isotope Distribution Calculator* (IDCalc, by Michael J. MacCoss, Department of Genome Sciences, University of Washington). Besides [M-H]^−^ ions, [M-H + NaCl]^−^ and [M + Cl]^−^ ions were detected. Their amount especially depended on the Cap Exit voltage and the performance of the instrument and therefore varies *interday*. For the evaluation, only the [M-H]^−^ ions were considered. Any impact of adduct formation was considered and is discussed.

#### Application of the optimized MS parameters to *O*-MeOEt-*O*-Me-celluloses

The optimized measurement parameters were applied to methoxyethylated MC1 and MC2 as well as to a methylated HEMC. Sample preparation consisting of peralkylation and partial hydrolysis was carried out as described in [19] and subsequent *m*ABA labeling as described in [11]. To determine the substituent distribution, the samples were diluted to a concentration of 0.05 mg mL^-1^ (90/10 ACN/H_2_O; v/v) and infused by ESI-IT-MS by syringe pump at a flow rate of 200 μL h^-1^. For the general parameters see *Instrumentation*. The spectra were recorded for DP2-4 using the selected parameters (see ESM section H, Table S7), and the usually applied standard parameters (TM1000; Trap Drive Level 100 %; Compound Stability 1000 %; Cap Exit -280 V; Oct 2 DC -2.7 V; Oct RF 200 Vpp, TD 99.6). Evaluation of the substituent profiles recorded with the selected parameters is described under *Application of the optimized instrumental settings to methoxyethylmethyl-cellulose*.

## Results and discussion

### Preparation of equimolar *O*-MeOEt_m_*O*-Me_n_ cellobiose mixtures (AB, BC, CD) and reference data analysis

In order to study sources of discrimination in the quantitative ESI-IT-MS analysis of methoxyethyl oligosaccharides derived from HEMC and HEC, effects during the ionization, ion transfer, and ion storage were investigated with defined cellobiose ether standards. These compounds with a defined number of Me and MeOEt groups were obtained from the corresponding cellulose ethers.

First, the standard solutions of the *m*ABA-labeled cellobiose ethers A, B, C, and D (for the structures see Fig. 2) were prepared at a concentration of about 4 ∙ 10^−4^ M. The exact concentration of the compound of interest was determined by HPLC-UV-MS based on the UV signal, whereas the MS signal was used to evaluate the peak purity. As products of multistep syntheses, such compounds always contain minor amounts of other constituents, and due to chain degradation during etherification reactions, terminal 4-*O*-alkyl glucosyl units were also present. The solutions of the analytes A and D showed minor signals of underalkylated oligosaccharides of DP3 as well as terminal 4-*O*-alklated units of DP1. For the mixed cellobiose, ethers B and C in addition tiny signals of regioisomers could be detected by the extraction of ion chromatograms with the respective *m/z* but not in UV. The contributions of these regioisomers were taken into account in the quantitative evaluation according to their ion intensity relative to the target compounds, since highly reliable reference data of the concentration of the analytes of interest are essential for our quantitative MS study. Based on the analyte concentrations of the standard solutions, equimolar binary mixtures of A + B, B + C, and C + D were prepared. Their total concentration was determined in the same way, and the molar ratio of the two respective analytes was calculated. The MR is defined as the ratio of the higher to the lower methoxyethylated cellobiose, i.e., B:A, C:B, and D:C, respectively. The results are shown in Table 1.

**Table 1:** Absolute concentrations of binary standard solutions and molar ratio (MR) of B/A, C/B, and D/C of DP2, determined by HPLC-UV-MS (*n* = 6) and standard deviation (SD); individual and total concentrations 1 ∙ 10^−4^ M; for structures of A, B, C and D, see Fig. 2. The concentrations of B and C include their regioisomers

			Concentration c∙10^−4^ M			
Binary	AB		BC		CD	
Mixture	A	B	B	C	C	D
DP2	0.40 ± 0.01	0.40 ± 0.01	0.51 ± 0.01	0.51 ± 0.01	0.99 ± 0.01	0.97 ± 0.01
Total	0.79 ± 1.74 %		1.01 ± 1.86 %		1.96 ± 1.23 %	
MR	0.990 ± 0.011 (±1.12 %)		0.992 ± 0.005 (±0.52 %)		0.982 ± 0.006 (±0.57 %)	

### Influence of the Trap Drive on the Ion storage

As mentioned in the Introduction, for the determination of the exact MR of analytes, it has to be ensured that there are not any discrimination effects during the ionization, the ion transfer, the mass analysis, and the detection process. The ESI-IT-MS used in this study is equipped with a Daly detector. On the one hand, the number of the emitted secondary ions depends on the speed of the ions and thus — at the same kinetic energy and charge — on their mass [23]. From this, it is estimated that the heavier compound of the binary mixtures is about 6–8 % slower compared to the lighter one. On the other hand, the dead time of an electron multiplier also depends on mass, but with different tendency, promoting overestimation of heavier ions due to shorter dead times [24, 25]. Based on this knowledge, we assume that the effect of the detector can be neglected compared to other devices.

In a recent study regarding potential discrimination effects in the ESI-IT-MS analysis of methylcellulose-derived Me/Me-*d*_*3*_ COS [19], we described how the maximum intensity of a particular analyte ion depends on the selected TD value (specifying the amplitude of RF voltage at the IT). The higher the *m/z* and the kinetic energy, with which the analyte enters the trap, the stronger the applied RF field (TD) must be for optimal storage [26]. This can be explained by the way an IT works as has been outlined in more detail in Ref. 19.

Consequently, the ion trap is crucial with respect to potential bias in relative quantification by IT-MS. Even in the case of binary mixtures of Me/Me-*d*_*3*_ COS, the TD values at maximum intensity of the isotopologs varied, but the discrimination effects were low due to the small ∆ *m/z* (DP2, 18; DP6, 54) [19]. In the case of HEC and HEMC, however, the ions to be analyzed cover a mass range ∆ *m/z* of 264 (*m/z* 546–810) already for DP2. Therefore, we designed the experiments differently and subdivided the mass range of Me/MeOEt DP2 into segments of ∆ *m/z* 88 with four representatives A, B, C, and D, which were labeled with *m*ABA in order to overcome differences in sodium complexation (Fig. 2) (AB, *m/z* 546–634; BC, *m/z* 634–722; CD, *m/z* 722–810). These analytes were measured at a total concentration of 10^-6^ M as [M-H]^−^ in negative mode (in the following, also negative voltages will be given unsigned).

To study the effect of TD on relative quantification, we recorded the ion intensities of the two analytes of each binary mixture in dependence on TD for different Oct 2 DC voltages (1.74, 2.00, 2.24, 2.48, and 2.70 V). In contrast, the Cap Exit and the RF voltage of the octopoles proved to have no influence on the kinetic energy in the transport direction at the trap entrance and thus no impact on the TD value at maximum intensity. Therefore, all other measurement parameters were chosen as common in the analysis of HEC and HEMC (for ESI s. *Materials and methods*; MS: Cap Exit 280 V, skimmer 40 V, Oct 1 DC 8 V, Oct RF 200 Vpp).

**Fig. 3:**
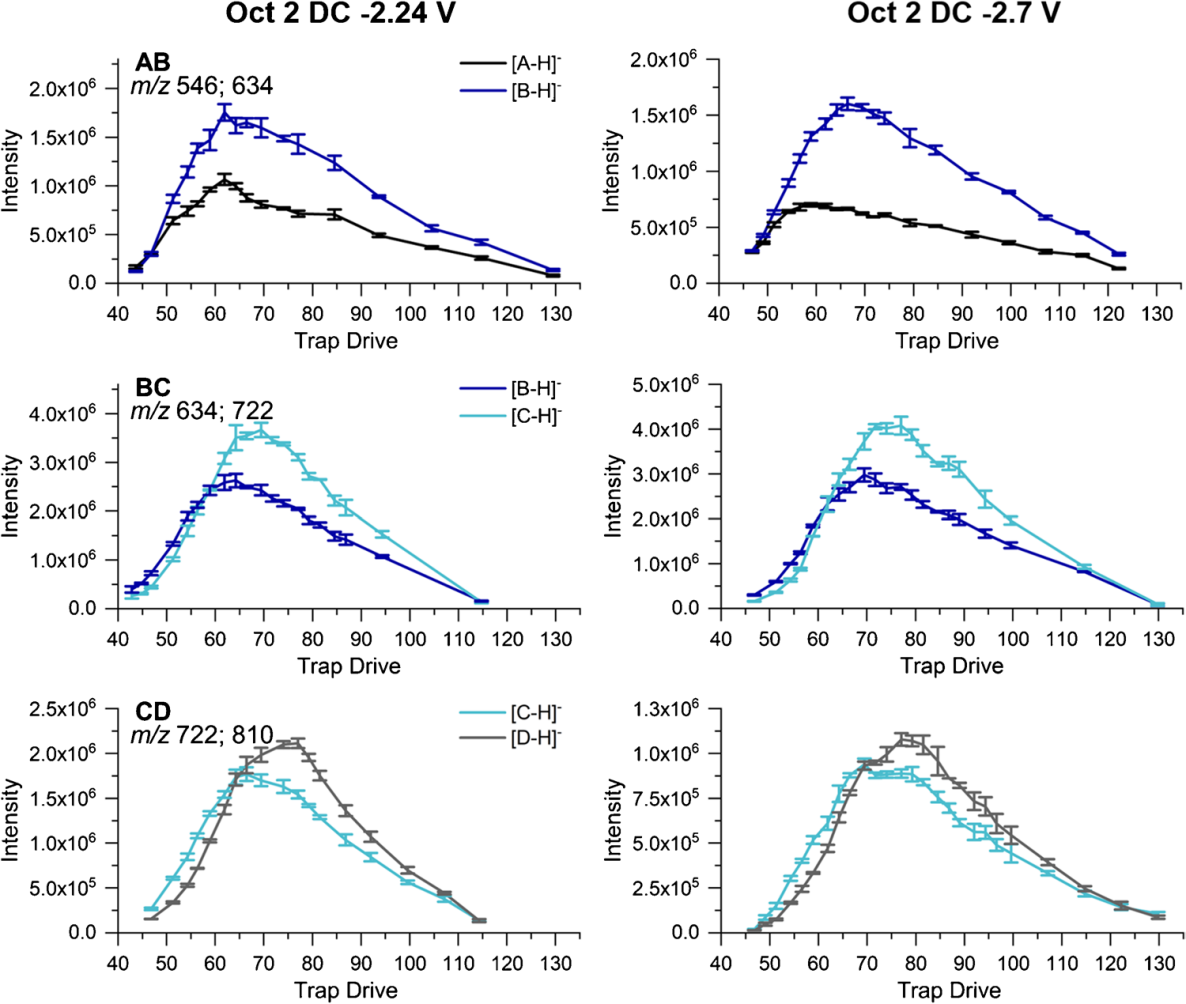
Absolute intensities recorded for the binary mixtures of *m*ABA-labeled cellobiose derivatives (AB, BC, CD, see Fig. 2) at a total concentration of 1 ∙ 10^−6^ M in ACN/H_2_O (90/10 v/v) by ESI-IT-MS (syringe pump infusion) at various TD (RF amplitude of the ring electrode) and Oct 2 DC voltages; Cap Exit is fixed at −280 V. Data are corrected for the exact MR according to the reference data given in Table 1 in order to represent an equimolar mixture; *n* = 3. For further measurement parameters, see *Material and Methods*

Figure 3 shows the results for two examples for each of the three binary mixtures, measured at Oct 2 DC 2.24 and 2.70 V, respectively. The intensities were normalized to an exact equimolar binary mixture. As can be seen, the intensity of the respective analyte ion steeply increases with TD, passes through a maximum (TD(I_max_) = TD_max_), and then slowly flattens out. The curve for the heavier analyte is always shifted to higher TD. This results in an IR/MR (intensity ratio/molar ratio) of < 1.0 at low TD before the point of intersection, steadily rising with increasing TD, and a slight decrease beyond TD 90/100 (s. ESM section B, Fig. S3).

**Fig. 4:**
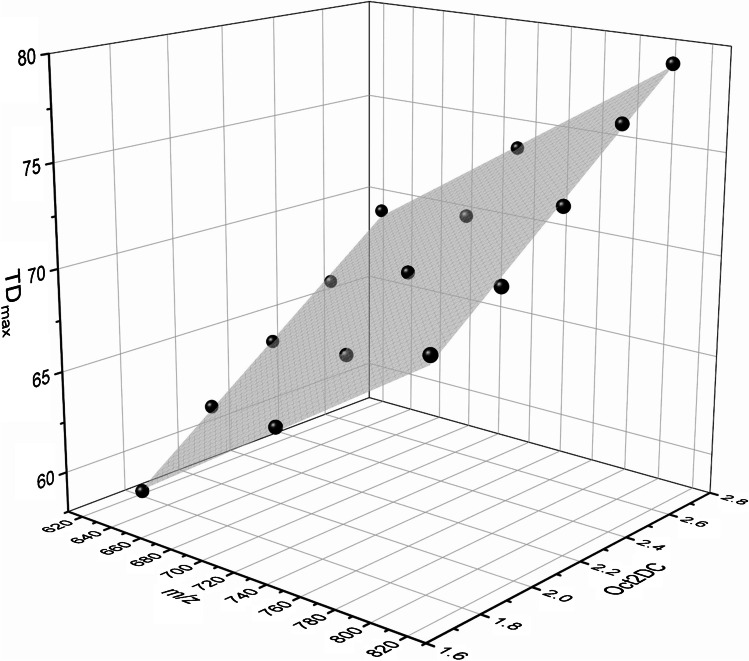
Dependence of TD_max_ (RF amplitude of the ring electrode) on *m/z* and Oct 2 DC, obtained from the experimental measurement data of the binary mixtures of *m*ABA labeled cellobiose derivatives (AB, BC, CD, see Fig. 2). *TD*_*max*_ = 0.0567 ∙ (*m*/*z*) + 10.52 ∙ *Oct* 2 *DC* + 4.69 (*R*^2^ 0.9974)

TD_max_ depends on the mass (*m/z*) of the analyte and the applied Oct 2 DC voltage. Based on all data recorded at various Oct 2 DC voltages, a correlation between *m/z* of the analyte, the applied Oct 2 DC voltage, and TD_max_ was established (Fig. 4, Equation 1):

1 $${TD}_{max}=0.0567\cdot \left(m/z\right)+10.52\cdot Oct\ 2\ DC+4.69$$

TD_max_ values were extracted from the manually fitted I(TD) curve. Due to this procedure and the measurement inaccuracy of the ion trap, an error of ± 1 of TD_max_ is estimated. For compound A, in some cases, the course of ion intensity was too broad to identify TD_max_ (compare Fig. 3 binary mixture AB at 2.7 V). Therefore, the data of A were not included in the correlation. *R*^2^ for the 3D correlation was 0.9974. From Equation 1, it can be seen that the TD_max_ values of compounds A, B, C, and D, each pair with a difference of ∆ *m/z* 88, differ by about 5 at the same Oct 2 DC voltage. It should be mentioned here that the exact values are susceptible to changes in the system that cause the ions to enter the trap with a different kinetic energy, e.g., deposits in ion optics (and ion trap) and changes in the vacuum stages (because of service maintenance).

In summary, the chosen TD value has a strong impact on a relative quantification and thus is a potential source of bias, if a relative quantification is required over a wide mass range. This discrimination can only be minimized by measuring at optimized values but not completely excluded. If no further discrimination occurs during the entire MS analysis, one would expect equal ion intensities of the analytes at their individual TD_max_. However, the intensity of the higher methoxyethylated COS was always larger at its TD_max_ (Fig. 3), indicating other sources of bias. Therefore, in the next step, the influence of Oct 2 DC and Oct RF on a relative quantification was studied. In order to be able to measure with highest accuracy possible, the midpoint of the two individual TD_max_ of the compounds of the respective binary mixtures was selected as TD-setting for the following measurements.

### Influence of octopole voltages on ion transportation

The two octopoles of the ESI-IT-MS (Fig. 1) operate as transport and focusing units. Compared to quadrupoles which are mainly used as mass filters, they show more diffuse ion stability limits and less defined *m/z* cut off and thus enable a better transmission over a wide *m/z* range [27]. As with the ion trap, the ions can only pass through multipoles if they move on stable ion trajectories. The highest ion transmission efficiency is expected at DC= 0 (only RF-mode), however, in order to reduce noise and enhance sensitivity and speed, the Oct 2 DC voltage is set to a minimum of 1.70 V, according to the manual. For more detailed information, how the applied DC and RF voltage effect stable ion trajectories the reader is referred to the literature [28].

**Fig. 5:**
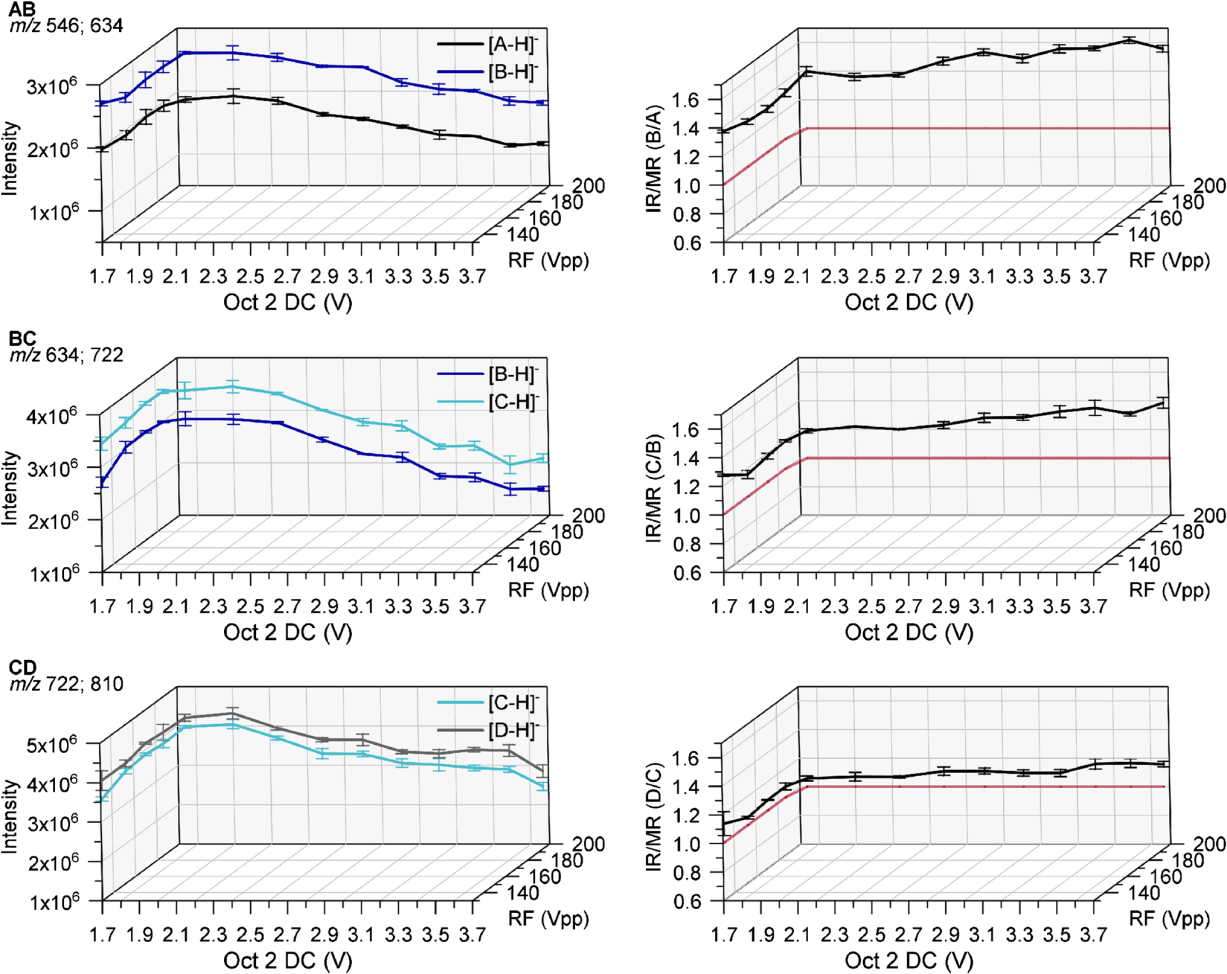
Left: absolute intensities recorded for the binary mixtures of *m*ABA-labeled cellobiose derivatives (AB, BC, CD, see Fig. 2) at a total concentration of 1 ∙ 10^−6^ M in ACN/H_2_O (90/10, v/v) by ESI-IT-MS (syringe pump infusion) at various negative Oct 2 DC and Oct RF voltages and at appropriate TD (midpoint of TD_max_ of the analytes according to equation 1), Cap Exit -280 V. Right: intensity ratio (IR). Data are corrected for the exact molar ratio (MR) according to the reference data given in Table 1 to represent an equimolar mixture; *n* = 3. For further measurement parameters, see *Materials and Methods*

As shown above, TD_max_ is shifted to higher values when Oct 2 DC is raised, since ions will enter the trap with higher kinetic energy. In order to study potential discrimination during ion transportation through the octopoles, we stepwise varied the Oct 2 DC and Oct RF voltages (based on the values suggested for a given TM in the *smart mode*). At the same time TD_max_ was adapted according to Equation 1. The midpoint TD_max_ obtained for the two analytes of a particular mixture was applied, respectively (s. ESM section C, Table S1). Thus, the results displayed in Fig. 5 in a 3D plot do not only show the dependence of the signal intensities on Oct 2 DC but on correlated Oct 2 DC and TD variation. According to the octopole management in the *smart mode*, we first increased the Oct RF voltage from 131 to 200 Vpp at constant Oct 2 DC of 1.74 V and subsequently increased Oct 2 DC up to 3.67 V at constant Oct RF voltage of 200 Vpp.

On the *yz-*plane on the left side of the graphics in Fig. 5, the ion intensities are shown with increasing RF voltage from 131 to 200 Vpp at a constant Oct 2 DC voltage of 1.74 V. In agreement with our previous study on Me/Me-*d*_*3*_ COS [19], there is no significant impact of Oct RF at constantly low Oct 2 DC on signal intensity and IR/MR, displayed in the right column. For RF 200 Vpp and Oct 2 DC 1.74 V, an IR/MR of 1.40 ± 0.03 is obtained for the mixture AB; for BC, it is 1.19 ± 0.01 and for CD 1.06 ± 0.02. The deviation from the real MR of 1.0 was the larger, the lower the *m/z* range. However, it should be noticed that all these measurements were performed at Cap Exit 280 V. For the Me/Me-*d*_*3*_ COS, we had observed that Cap Exit can cause diverging intensities at lower *m/z*. Thus, not the absolute MR values, but the robust and sensitive region and the general impact on the relative intensities has been deduced from these experiments. Also discrimination effects during the ionization have still to be considered.

On the *xy*-plane of the diagrams in Fig. 5, the subsequent increase of Oct 2 DC at a constant RF of 200 Vpp is displayed. As can be seen, for all analyte pairs, the highest and nearly constant intensities were observed in the Oct 2 DC range 1.74 V to 2.24 V or 2.0 V (mixture CD). At further increase of Oct 2 DC, both intensities decrease approximately equally. Hence, the IR/MR remained constant up to Oct 2 DC of 2.24 V and then slowly increased.

In conclusion, as expected, the octopole settings have significantly less impact on the finally detected relative ion intensities than the RF voltage at the ion trap, as long as TD is varied accordingly. At Oct 2 DC voltages up to 2.24 V (RF 200 Vpp) and at the different Oct RF voltages at constant Oct 2 DC 1.74 V, no selective transport to the mass analyzer is observed for our analyte pairs with ∆ *m/z* 88. The difference in ion yields and IR/MR remains constant, the difference being more pronounced for the analyte mixtures of lower *m/z* (due to Cap Exit, see below). At Oct 2 DC > 2.2 V, the absolute intensities for both analytes decrease since the region of stability become narrower [28], while IR/MR increases. Therefore, an Oct 2 DC voltage of 1.74 V and an Oct RF voltage of 200 Vpp were selected as settings for all mixtures. Thus, the source for the intensity differences in spite of equimolarity cannot be eliminated by appropriate octopole voltages but must originate from an earlier step of ion formation or transport which will be studied in the following steps.

### Influence of Cap Exit and Skimmer voltage on ion transportation

The ions formed in the electrospray ionization unit at atmospheric pressure reach the first low pressure area via a glass transfer capillary. Nitrogen is used as dry gas, so that clusters of solvent and ions can be destroyed by desolvation and collision. Page et al. studied the effect of various parameters, such as temperature or length, on the transport efficiency of ions through the capillary [29]. Behind the capillary exit, the ions are transferred to the second low pressure area via a skimmer (see Fig. 1). There, clusters can also be destroyed by collision-induced dissociation (CID) due to the potential difference between the capillary outlet (nozzle) and the skimmer — the so-called nozzle skimmer CID. Depending on the potential differences and the analytes’ stability, dissociations of non-covalent complexes and even fragmentation of covalent bonds can occur [30–33]. Beside the pressure gradient, the voltage between capillary outlet (Cap Exit) and skimmer is the driving force for the ions towards the high vacuum area of the instrument.

**Fig. 6:**
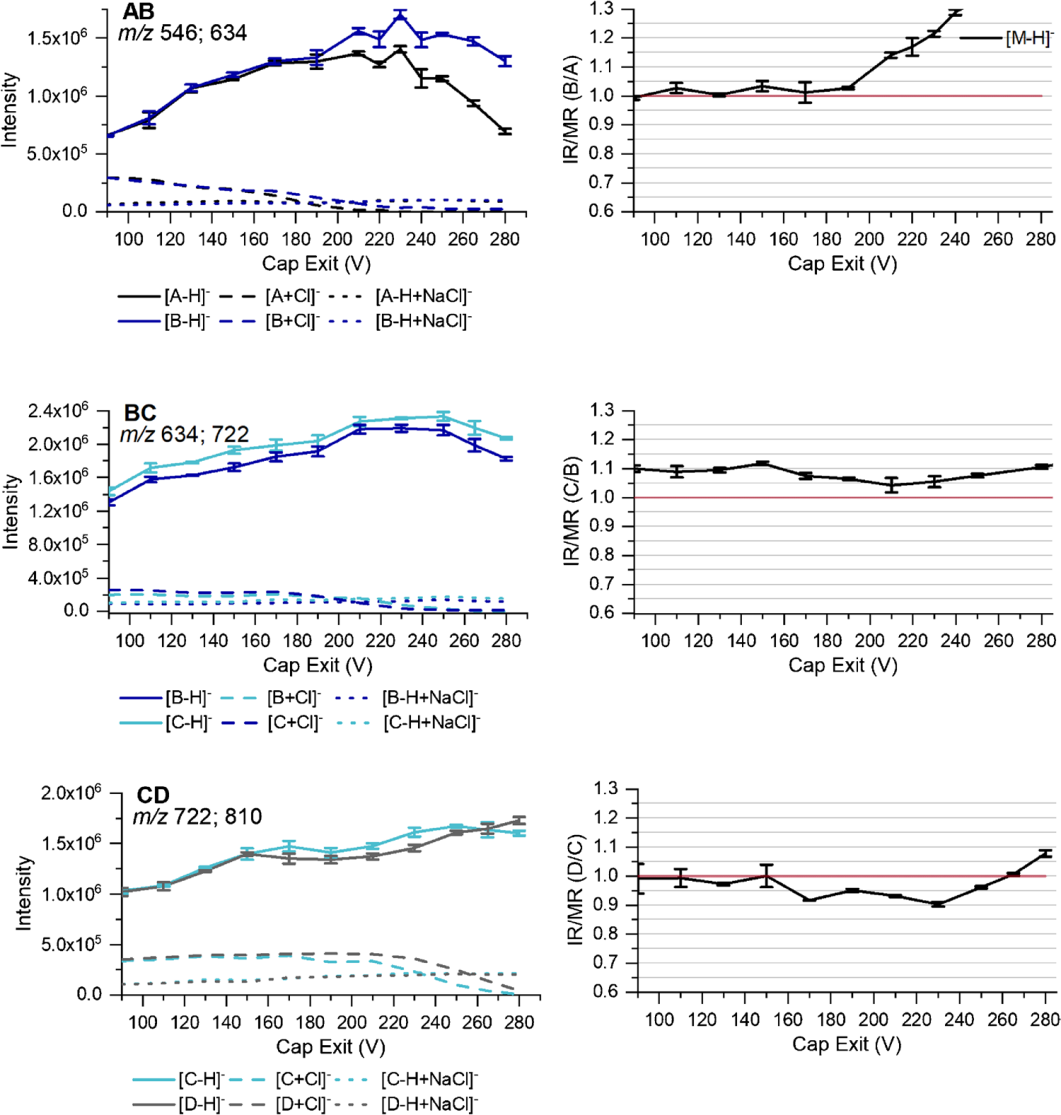
Left: absolute intensities recorded for the ions formed for the binary mixtures of *m*ABA-labeled cellobiose derivatives (AB, BC, CD, see Fig. 2) at a total concentration of 1 ∙ 10^−6^ M in ACN/H_2_O (90/10 v/v) by ESI-IT-MS (syringe pump infusion) at various Cap Exit voltages (negative). Right: IR/MR for [M-H]^−^-ions. Data are corrected for the exact molar ratio (MR) according to the reference data given in Table 1 to represent an equimolar mixture; *n* = 3. For further measurement parameters, see text and *Material and Methods*

All measurements presented above have been performed at Cap Exit 280 V. In the left column of Fig. 6, the influence of Cap Exit (negative values) on the ion intensities is shown, while in the right column, the IR/MR values are displayed. The skimmer was kept constant at 40 V during these measurements. In addition to the intensities of [M-H]^−^ ions, those of chloride adducts [M+Cl]^−^ and cluster with NaCl [M-H+NaCl]^−^ are presented. The latter could be differentiated from overalkylated by-products (1 additional Me (O-4) and 1 Me substituted by MeOEt or one additional MeOEt (O-4) also correspond to a mass increment of +58) by their chloride specific isotopic pattern. Due to the many oxygen atoms, the analytes very well form complexes with salts, which have not been considered in the evaluation so far.

For all three binary mixtures, rising intensities of [M-H]^−^ ions are observed with increasing Cap Exit voltage, on the one hand because of the increasing potential gradient between Cap Exit and skimmer (improved transport) and on the other hand because of the loss of HCl from chloride adducts [M+Cl]^−^ by CID. This ion species was found to be slightly more stable for the higher methoxyethylated compound in the binary mixtures, thus requiring a higher Cap Exit voltage for dissociation of both of them. In contrast, the NaCl cluster proved to be particularly stable and remained at a constant ratio related to the target ion but at equal extent for both analytes in a particular measurement (for corresponding data s. ESM section D, Table S2-S4). After going through a maximum, the intensities of the target ions finally decrease again. The maxima are shifted with increasing *m/z* to the higher Cap Exit area. For A, the maximum is reached in the range 170–210 V, while for B, it is observed at 210–250 V. The intensity of C reaches its highest value at about 250 V, whereas the intensity of D is still increasing at the maximum Cap Exit of 280 V. While the analytes of BC and CD behave very similar, the drop of intensity for A is so pronounced that A and B start to diverge above 180 V. Consequently, the IR/MR, shown in the right column of Fig. 6, being close to 1.0 up to 180 V, steeply increases beyond this mark. This is the reason why in the measurements performed at Cap Exit 280 V, IR for AB is as high as 1.4. For BC, courses of intensities are nearly parallel. IR/MR is between 1.05 and 1.10 over the entire Cap Exit range. Finally, CD shows IR/MR between 1.0 and 0.9 until the intensities start to diverge at about 270 V. The higher risk for bias for smaller ions with lower *m/z* (< 600) has also been observed in our study on Me/Me-*d*_*3*_ COS for DP2 [19]. Due to their lower mass, these ions gain higher velocities which enhances the probability to collide with other ions or strike the capillary wall. Furthermore, the more rigid permethylated cellobiose has less possibilities for dissipation of the collision energy compared to the MeOEt-ether with overall six additional free rotating linkages. Since NaCl cluster were stable and did not affect the IR, the elimination of HCl from [M+Cl]^−^ thus generating target ions [M-H]^−^ was considered for the choice of Cap Exit. Optimal would be the full destruction of these additional ions; the extent of which also varies with the performance of the instrument. Therefore, 250 V for BC and 280 V for CD were chosen. Under these conditions, the chloride adducts were almost fully destroyed. For the binary mixture of AB, it was more difficult. For B, the [M+Cl]^−^ clusters were completely destroyed at 230 V; however, at this point, the intensities of AB started to diverge. Therefore, we chose 150 V; at this value HCl clusters were still observed but at very similar ratio for both analytes. In Table 2, the final measurement parameters as well as the IR/MR obtained under these conditions are presented.

**Table 2:** Finally selected measurement parameters for the quantitative analysis of *m*ABA-labeled binary mixtures of cellobiose derivatives (AB, BC, CD, see Fig. 2) and IR/MR found under these conditions. IR is defined as the intensity of the higher methoxyethylated cellobiose, divided by that of the lower methoxyethylated one: B/A, C/B, and D/C, respectively. Skimmer voltage 40 V; Oct 1 DC 8 V; Oct RF 200 Vpp, *n* = 3. All voltages are negative voltages

Mixture	*Trap Drive*	Oct 2 DC [V]	Cap Exit [V]	IR/MR
AB	56.5	1.74	150	1.03 ± 0.02
BC	61.5	1.74	250	1.08 ± 0.01
CD	66.5	1.74	280	1.08 ± 0.01

In summary, it was shown that Cap Exit can cause pronounced bias in a relative quantification, especially at low *m/z* and different chemical stabilities. It has to be considered carefully, when measurement parameters are defined. Additional adduct ions and cluster formation do not necessarily cause a bias when only [M-H]^−^ ions are considered, as long as these ions are formed to the same extent for both compounds. Yet, cluster destruction is preferred due to potential signal interference in more complex samples.

### Concentration-dependent ESI-MS measurements

So far, only discrimination effects during ion transportation and storage have been considered, assuming that the analytes of the binary mixtures have the same ionization efficiency. In our recent study of the binary mixtures of Me/Me-*d*_*3*_ COS [19], no discrimination was observed during ionization. However, these were isotopologs. This time, we look at compounds that differ in the number of CH_3_O(CH_2_)_2_ and CH_3_ groups and thus possibly in surface activity and complexation ability as well. The latter source of bias in ionization was eliminated by labeling the compounds with *m*ABA and measurement of the corresponding anions in the negative mode.

However, from the literature, it is known that the ions that are located on the droplet surface have a higher chance of getting into the next droplet generation and consequently to reach the gas phase. The equilibrium constant *K* (equation 2) is a good indicator of surface activity [34]:

2 $${K}_A=\frac{{\left[{A}^{+}\right]}_s\kern0.5em {\left[{X}^{-}\right]}_i}{{\left[{A}^{+}{X}^{-}\right]}_i}$$

It depends, among other things, on the polarity, the charge density, and the basicity of the analyte. Analytes (A^+^) with a high *K*-factor prefer to be located on the droplet surface (s) and will be capable of carrying a large amount of the excess charge, whereas analytes with a low factor tend to be located inside the droplet (*i*) and be paired with counter ions (*X*^−^) [35].

Below the saturation point, two charged analytes should show equal sensitivity and linear increase of intensity with concentration, as long as the *K* factors are not too different. At the saturation point (c~10^−5^ M), however, a competition for the limited space arises, and the analyte with the higher surface activity will displace the other one from the surface and thus suppress it [34, 36].

We made use of this effect to find out whether any bias during ESI can be excluded. Therefore, we performed concentration-dependent measurements of the binary mixtures in the range of approximately 10^−9^ M to 10^−4^ M (total concentration) at the optimized measurement parameters (Table 2). A divergence of the ion intensity curves occurring at the saturation point, and thus an abrupt increase or decrease of IR/MR, would indicate discrimination in the ESI process.

**Fig. 7:**
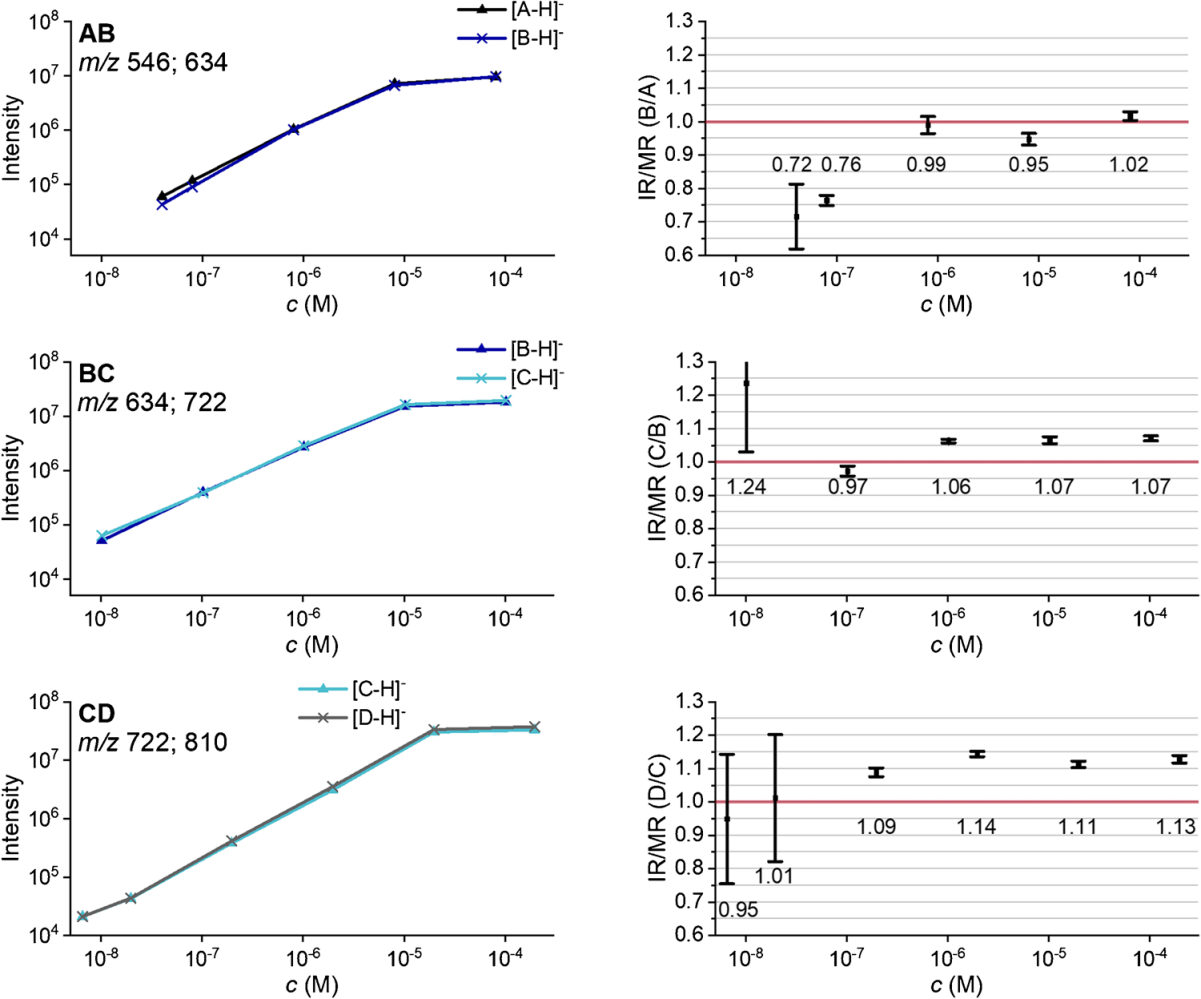
Left: absolute intensities of the serial dilution (total concentration) of the binary mixtures of *m*ABA-labeled cellobiose derivatives (AB, BC, CD, see Fig. 2) in ACN/H_2_O (90/10 v/v) by ESI-IT-MS (syringe pump infusion). Right: calculated intensity ratio (IR). Data are corrected for the exact molar ratio (MR) according to the reference data given in Table 1 to represent an equimolar mixture. Further measurement parameters are given in Table 2; *n* = 5

Figure 7 shows the results. For all mixtures, the double logarithmic plots show a linear increase of the intensities with concentration with a flattening above 10^−5^ M due to saturation, but no ion suppression effect is visible. Below a total concentration of 10^−7^ M, signal-to-noise ratio (S/N) was too poor to get reproducible results. As displayed in the right column of Fig. 7, for the binary mixture AB, IR/MR was close to 1.00, and for BC and CD, it was slightly above this real ratio (1.07 and 1.12, respectively).

To check the reproducibility of the results, the measurements for the concentrations 10^−6^ to 10^−5^ M were repeated on 2 further days; for mixture CD, we also recorded 10^−7^ M (s. ESM section E, Table S5). The *intraday* as well as the *interday* standard deviations were between ±0.01 (1 %) and ±0.03 (3 %).

Finally, the normalized intensity ratio IR/MR was determined from all measurements, which were recorded during the studies under the later specified appropriate measurement parameters and for a concentration between 10^−6^ and 10^−4^ M; for the mixture CD, we also considered 10^−7^ M (Table 3). IR/MR was calculated as weighted average, and the external standard deviation was determined (s. ESM section F). The determination of the uncertainty budget was carried out according to Type A evaluation, taking into account both, the standard deviation of the HPLC reference method and the MS measurements. A *t*-distribution was assumed and a confidence interval of 95.0 %.

**Table 3:** IR/MR of the binary mixtures AB, BC, and CD, given with expanded uncertainty U (95 % confidence). IR is defined as the intensity of the higher methoxyethylated cellobiose, divided by the lower methoxyethylated one: B/A, C/B, and D/C, respectively. Ratios are a weighted average of 8–12 values per binary mixture, which were received each from triplicate and quintuplicate measurements

	AB	BC	CD
**IR/MR**	0.985 ± 0.014 (1.4 %)	1.074 ± 0.006 (0.6 %)	1.114 ± 0.006 (0.6 %)

The experimentally determined IR increased from 0.985 ± 0.014 (B/A) to 1.074 ± 0.006 (C/B) further to 1.114 ± 0.006 (D/C) for the mixtures. There might be various reasons for the systematic deviation from the real ratio of 1.0. On the one hand, the determination of TD_max_ is critical. The intensity curves are not symmetrical. They rise steeply but fall more slowly. This means that measurement at $\frac{\left({TD}_{\max (1)}+{TD}_{\max (2)}\right)}{2}$ can only approximate the optimum TD, which should be closer to analyte 1. Furthermore, small uncertainty of the slope and the axis intercept of Equation 1 will cause TD_max_ deviations with a significant impact on IR. Thus, a shift of TD_max_ of ∆ ± 1 causes a change in IR/MR of about 0.03–0.04. On the other hand, the chosen Cap Exit value for the mixtures BC (250 V) and CD (280 V) might be too high. Due to the cluster, we chose the highest acceptable value. However, this is almost at the threshold of CID for the lighter compound of the mixture. Consequently, too high values would be obtained for IR/MR.

This discussion of error sources brings up a general crucial aspect. We strived towards finding instrumental settings, at which the analytes can be measured without discrimination. As is obvious from the development of IR/MR with TD, this regime is not very robust. An alternative approach could be to look for a robust area, where IR/MR does not change significantly with TD. This behavior is found at higher TD values, above TD 77 for all mixtures measured at the optimized Cap Exit and Oct settings (ESM section G, Fig. S4). However, here, the IR/MR is above 1.2 for all three mixtures. Consequently, in case of measuring under these conditions, correction factors must be applied. Since in a real sample, further MeOEt/Me derivatives exist with *m/z* between our model compounds, a correction factor for them must also be estimated. As we will see in the next chapter, dealing with the application of our results to permethoxyethylated MC1 and 2 as well as permethylated HEMC, this alternative is not superior to our approach of discrimination-free measurement without correction.

### Application of the optimized instrumental settings to methoxyethylmethyl-cellulose

**Fig. 8:**
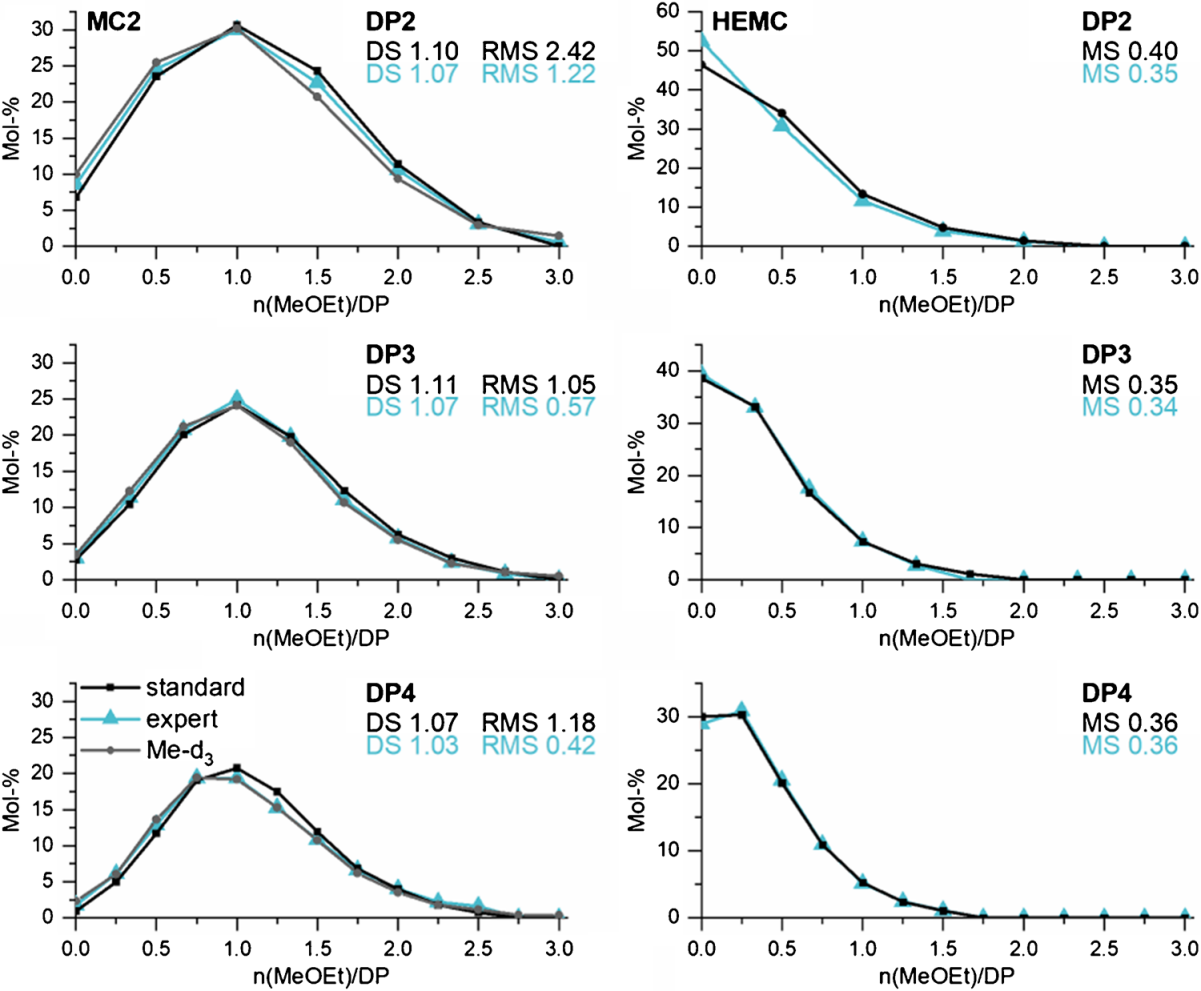
Methoxyethyl distribution of MeOEt/Me celluloses (DP2-4) obtained by ESI-IT-MS of *m*ABA-labeled COS, derived from permethoxyethylated MC2 (DS_MeOEt_ 1.05) (left column) and permethylated HEMC (Zeisel-MS_HE_ 0.35) (right column) measured by syringe pump infusion under standard conditions (TD 99.6; Cap Exit -280 V; Oct 2 DC -2.7 V; Oct RF 200 Vpp) and expert conditions (s. ESM section H, Table S7). For MC2, the distribution obtained after perdeuteromethylation (Me-*d*_*3*_) according to [19] is used as reference data. The deviation of the results obtained under expert and under standard conditions from these reference data is given as root mean square (RMS). *n* = 3

With the knowledge of the systematic study, we investigated the more complex COS mixtures obtained from MeOEt/Me celluloses. The optimal parameters found for the binary mixtures of DP2 were applied to determine their hydroxyethyl distribution on the oligomeric level. Based on Equation 1, it was also possible to establish suitable parameters for DP3 and DP4 (TD calculated, Cap Exit 280 V, Oct 2 DC 1.74 V, Oct RF 200 Vpp). As we have seen, it is not possible to measure the whole mass range of a particular DP at one single TD setting without discrimination effects in the ion trap. Therefore, the mass range was divided into smaller *m/z* segments. Finally, the intensities are interrelated to gain the entire distribution profile. The measurement settings are presented in the ESM (section H, Table S7). First, we applied these to permethoxyethylated MC1 (DS_Me_ 1.29) and MC2 (DS_Me_ 1.95) as reference polymeric material, since their substituent distribution has been determined after perdeuteromethylation by ESI-IT-MS without discrimination [19]. After permethoxyethylation, the MCs shall have a DS_MeOEt_ of 1.71 (MC1) and 1.05 (MC2), respectively, and the MeOEt pattern will be complementary to the original methyl pattern. The samples were measured by syringe pump infusion, after partial hydrolysis and *m*ABA labeling in triplicate for each segment condition (s. ESM section H, Table S7). For the relative molar quantification of all constituents belonging to one DP, the IR calculated after noise and isotope correction were interrelated to the ratios of the next overlapping segment via the analyte of the overlapping area (for instance B in AB and BC). Finally, the molar portions of all constituents were normalized to 100 %. Figure 8 shows the methoxyethyl distribution obtained for MC2 (DS_MeOEt_ 1.05), DP2-4 (left column). The distribution, received under the so-called expert conditions, is compared with the results obtained under the standard conditions usually applied in the *smart mode* (TM1000; Trap Drive Level 100 %; Compound Stability 1000 %; Cap Exit 280 V; Oct 2 DC 2.7 V; Oct RF 200 Vpp, TD 99.6) and with the corresponding Me-*d*_*3*_ distribution for the perdeuteromethylated MC (reference Me-*d*_*3*_). As mentioned above, a control parameter of non-discriminatory measurement is the DS that should be in agreement with the average DS of the material and constant over the DPs (in case of tandem substitution the MS, respectively). This is fulfilled for the expert conditions. On average, a DS_MeOEt_ of 1.06 is obtained for MeOEt-MC2 which agrees well with the complementary DS_Me_ of 1.95. The DS found for the standard conditions, on the other hand, is not constant. It is generally too high and decreases from 1.10 (DP2) to 1.07 (DP4). For DP2, the overestimation is due to the unsuitable Cap Exit (280 V) voltage which discriminates the constituents of lower *m/z,* i.e., the higher methylated constituents, as discussed above. As second control parameter, the root mean square (RMS) value was calculated (s. ESM section H, equation (*S4*)), to express the overall deviation between the reference data (Me-*d*_*3*_) and the distribution obtained under expert and standard conditions, respectively. These values show that the expert conditions (RMS 0.42–1.22) were always superior to the standard conditions (RMS 1.05–2.24). Comparable results regarding DS and RMS were obtained for MC1 (s. ESM section H, Fig. S5).

In the right column of Fig. 8, the results for a HEMC (MS_HE_ 0.35) are presented, measured under expert and standard conditions. For both conditions, the experimentally determined MS_HE_ (0.34–0.36) was close to the average MS_HE_ of the material. Again, for DP2, the MS_HE_ found under standard conditions (0.40) was too high, due to the discrimination at high Cap Exit. In conclusion, the instrumental settings for DP2-4 as well as the segmental measurement and evaluation principle are suitable for determining the MeOEt distribution of permethylated HECs and HEMCs samples.

As mentioned above, the midpoint of TD_max_ is not a very robust regime. Therefore, we also measured DP2 of the two MeOEt-MCs and the permethylated HEMC at TD 77 where IR/MR for all mixtures was approximately constant (s. ESM section G). The IR/MR evaluated by a linear fitting of the robust areas is 1.234 (AB), 1.283 (BC), and 1.279 (CD) for TD 77. Since these values were very close and did not show a trend, we assumed a constant factor for the intensity increase with *m/z* (i.e., number of Me substituted by MeOEt). The average of the three IR/MR is 1.265 (∆ *m/z* 88). Consequently, each compound with one MeOEt group more is detected better than the previous component by a factor of 1.125 (√1.265). The corresponding correction factors for each compound of DP2 are given in the ESM (section G, Table S6). The further measurement parameters were analogous to the expert conditions, i.e., Cap Exit was varied (150, 250 and 280 V, see ESM section H, Table S7).

**Fig. 9:**
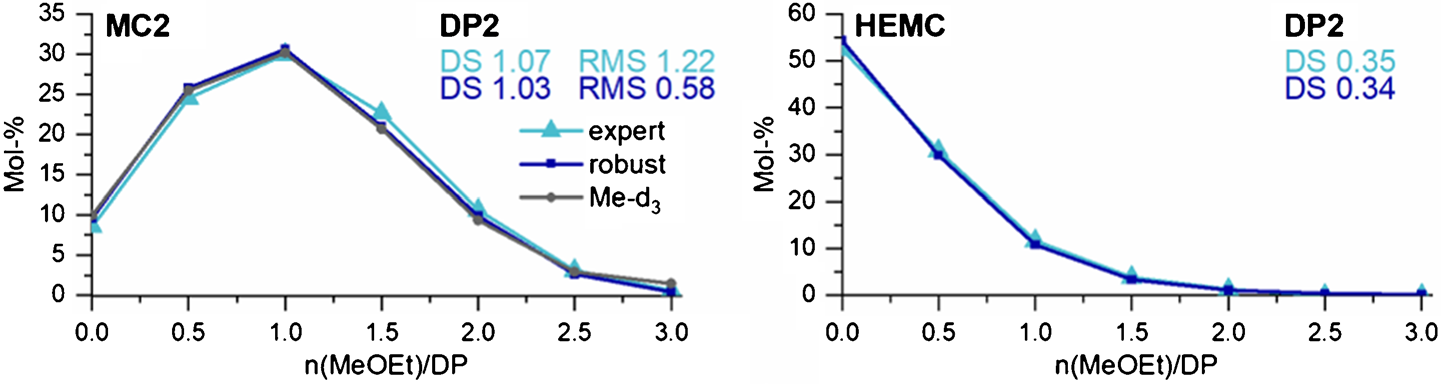
Methoxyethyl distribution of DP2 obtained by ESI-IT-MS of *m*ABA-labeled COS, derived from permethoxyethylated MC2 (DS_MeOEt_ 1.05) (left column) and permethylated HEMC (Zeisel-MS_HE_, 0.35) (right column) measured by syringe pump infusion under expert conditions (s. ESM section H, Table S7) and at a constant TD value (TD77, robust range) with correction of peak areas according to the intensity increase with MeOEt, given in Table S6 of ESM. Further measurements parameters were analogous to expert conditions. For MC2, the distribution obtained after perdeuteromethylation (Me-*d*_*3*_) according to [19] is used as reference data. The deviation of the results obtained under expert and robust conditions from these reference data is given as root mean square (RMS). *n* = 3

Figure 9 compares the methoxyethyl distributions of DP2 for MC2 and the HEMC obtained by segmental measurement under expert conditions and at a constant TD 77 and subsequent signal correction, respectively. The results for MC1 are shown in the ESM (section H, Fig. S6). For MC2 and HEMC under both measuring conditions, the DS and MS value agreed well with that of the sample. For MC2, RMS (related to the reference data for Me-*d*_*3*_) for the robust conditions (0.58) was even lower than for the expert conditions (RMS 1.22).

For MC1 (DS_MeOEt_ 1.71), on the other hand, the expert conditions were superior both in terms of DS_MeOEt_ (1.69) and agreement with the reference data (RMS 1.31). Under robust conditions, the DS_MeOEt_ was underestimated by 0.04 which was also reflected in the RMS of 1.87. Consequently, measuring at only one TD but of a robust range is a good alternative to the segmental measurement method working without signal correction. However, a disadvantage arises from the fact that measurement in the robust range cannot simply be extended to larger DPs. On the one hand, the robust range and the necessary correction factors cannot be predicted for higher DPs but must be experimentally determined. On the other hand, the robust range is far from the intensity maxima. Keeping in mind that partial hydrolysis produces a mixture with molar portions of COS, exponentially decreasing with DP (most probable distribution), it is evident that the measurements of the higher DPs would suffer from the expected loss of intensity in a robust area.

## Conclusion

The substituent distribution along and among the cellulose chains of hydroxyalkyl celluloses like HEMC or HEC requires a correct quantification of the molar ratios of all constituents with a particular number of MeOEt groups belonging to one DP. COS of various DP are obtained by partial hydrolysis after permethylation of HEMC or HEC, respectively. To overcome differences in sodium complexation, COS are labeled with *m*ABA and measured as [M-H]^−^. Nevertheless, a mass difference of Δ *m/z* 44 remains between the constituents of each DP. Therefore, a deeper understanding of how measurement settings affect a relative quantification is indispensable. With equimolar binary mixtures of defined cellobiose ethers with increasing number of methoxyethyl and decreasing number of methyl groups (Δ *m/z* 88, 2x MeOEt), potential sources of bias during ionization, ion transport, and storage have been studied.

No ion suppression effects were observed in concentration-dependent measurements above saturation. Regarding ion transfer, the choice of Cap Exit is especially crucial for low *m/z* analytes with less MeOEt residues which could dissipate the energy and thus protect against CID. Whereas a non-selective ion transport through the octopoles is possible at low DC voltages, the ion trap is particularly susceptible to discrimination due to its mode of operation (collection of ions at constant RF voltage). An equation describing the relationship between Oct 2 DC, *m/z*, and TD_max_ (TD at maximum intensity) was established from the experimental data and applied to calculate TD_max_ for higher DPs (larger COS). Under the optimized conditions, the IR of two cellobiose derivatives with a difference of two MeOEt, normalized to equal molarities, was determined to be 0.99–1.11 (SD, 0.6–1.4 %), respectively.

Based on the results of the binary mixtures of cellobiose, it was possible to find suitable measurement settings also for higher DPs. Due to the wide mass range to be analyzed, it is not possible to measure the constituents belonging to a particular DP at one single TD setting without discrimination in the ion trap. Therefore, the mass range was divided into smaller *m/z* segments. Data recorded at optimized instrumental adjustments without bias for overlapping narrow *m/z* segments were interrelated. Applications of the optimized segmental method to two well-investigated methoxyethylated MCs as polymeric reference material and finally to a HEMC with MS_HE_ 0.35 showed very good agreement with the reference data. If measured under standard conditions applied to cellulose ethers in ESI-IT-MS so far, only slightly larger deviations were observed but a systematic error for DP2. As an alternative, a method measuring at high TD in a robust area with subsequent signal correction worked for DP2 but cannot simply be transferred to larger COS.

The study therefore shows that beside isotopologous Me/Me-*d*_*3*_ COS ethers [19], even the reliable measurement of complex cellulose ethers with larger differences in chemistry and mass is possible, if the impact of instrumental settings is known and considered. Although time-of-flight (ToF) mass analyzers are less prone to discrimination [7] than ion traps, the more and more popular ESI-ToF instruments do not automatically solve the potential discrimination problem. Here, the instrumental settings controlling the ion transportation through the ion optics, the quadrupole as well as the collision cell and the transfer to the flight tube (transfer and pre pulse storage time) are also potential sources of bias and have carefully to be adjusted.

## Supplementary Information

Below is the link to the electronic supplementary material.

Supplementary file1 (DOCX 998 kb)
