# Supplementary material for: Impact of instrumental settings in electrospray ionization ion trap mass spectrometry on the analysis of O-methoxyethyl-O-methyl cellulose: a comprehensive quantitative evaluation
Source: Anal Bioanal Chem. 2022 May 3;414(16):4727–43. doi: 10.1007/s00216-022-04095-3 (PMC9174117; doi:10.1007/s00216-022-04095-3)
Supplement: Supplementary file 1 — Supplementary file1 (DOCX 998 kb) [file 216_2022_4095_MOESM1_ESM.docx]

*Analytical and Bioanalytical Chemistry*

## Electronic Supplementary Material

**Impact of instrumental settings in electrospray ionization ion trap mass spectrometry on the analysis of *O*‑methoxyethyl-*O*-methyl cellulose: a comprehensive quantitative evaluation**

Sarah Schleicher, Dominik Horoba, Philip Krafzig, Petra Mischnick*

Institute of Food Chemistry, Technische Universität Braunschweig, Schleinitzstr. 20, 38106 Braunschweig, Germany

*Corresponding author: Petra Mischnick, [p.mischnick@tu-braunschweig.de](mailto:p.mischnick@tu-braunschweig.de)

ORCID, Petra Mischnick: 0000-0002-8313-3313

1. **Preparation of the cellobioses standard compounds A, B, C and D**

Fully *O*-methylated (A) and fully *O*-methoxyethylated (D) standards were synthetized from cellulose acetate by alkylation. For the mixed cellulose ethers 2,3‑*O*‑methyl-6-*O*-methoxyethyl (B) and 2,3-*O*-methoxyethyl-6-*O*-methyl cellulose (C), 6-*O*-tritylcellulose was the starting material, which was alkylated in two steps. Before the second alkylation, the trityl protection group in position 6 was removed. Completeness of deprotection was checked by absence of aromatic vibrations by ATR-IR (example in Fig. S1).

Afterwards, the cellulose ethers were partially hydrolyzed and fractionated on a RP-C_18_ column. Since the cellooligosaccharide ethers (COS) are not UV-active, the retention time was checked using an ELSD detector, while the fractionation was carried out without a detector. Fig. S2 shows the HPLC-chromatogram of the 2,3-MeOEt-6-Me-COS.


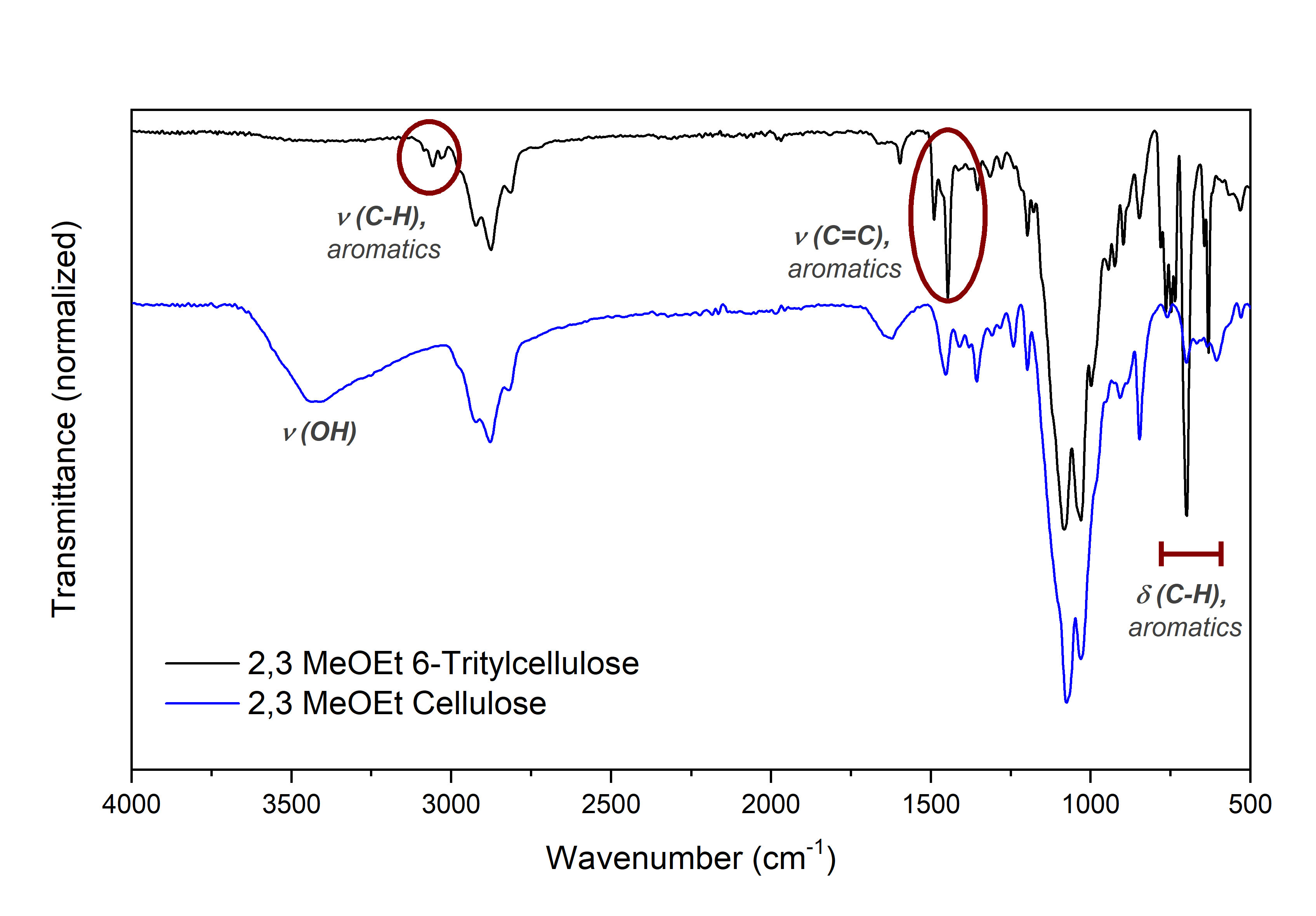


**Fig. S1** ATR-IR spectra of 2,3-MeOEt-6-trityl cellulose before (black) and after deprotection at *O*-6 (blue). Completeness of deprotection was checked by absence of aromatic vibrations


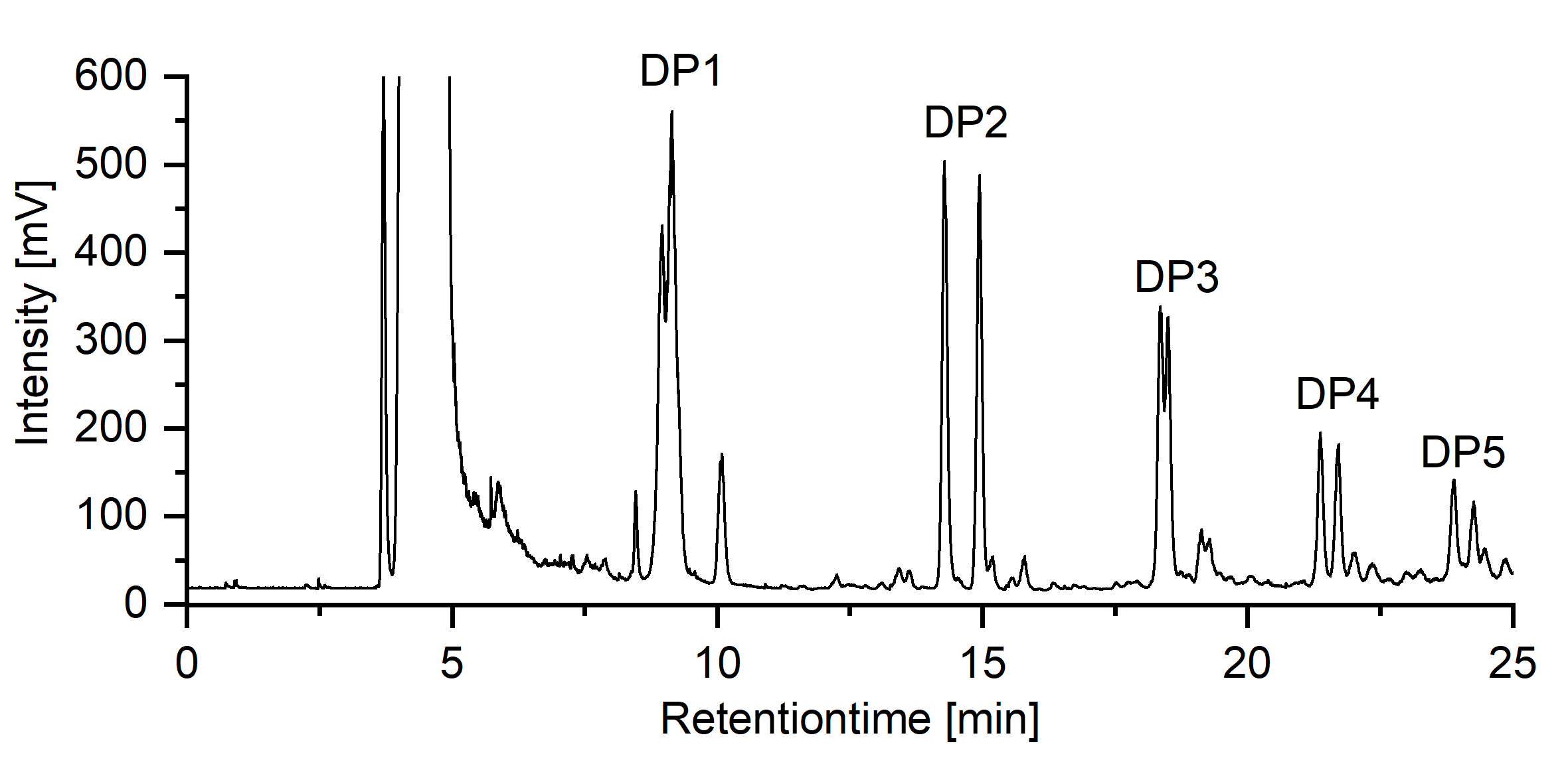


**Fig. S2** ELSD-chromatogram of a partial hydrolysate of 2,3-MeOEt-6-Me cellulose, seperated on a C_18_ column using a linear gradient of H_2_O + 1 % HOAc (A) and ACN+1 % HOAc (B) as mobile phase, starting with 80 % A to 0 % within 50 min, 1 mg partial hydrolysate was injected

1. **Influence of TD on IR/MR**

The *m*ABA-labeled binary mixtures of MeOEt/Me-cellobiose ethers AB, BC and CD (see Fig. 2, body text) were measured at different Trap Drive values (TD, measure for the RF amplitude of the ring electrode) for different Oct 2 DC voltages. In the body text the recorded absolute intensities for Oct 2 DC -2.24 V and -2.7 V are presented (Fig. 3). In Fig. S3 the corresponding IR/MR (intensity ratio/molar ratio) values are presented.


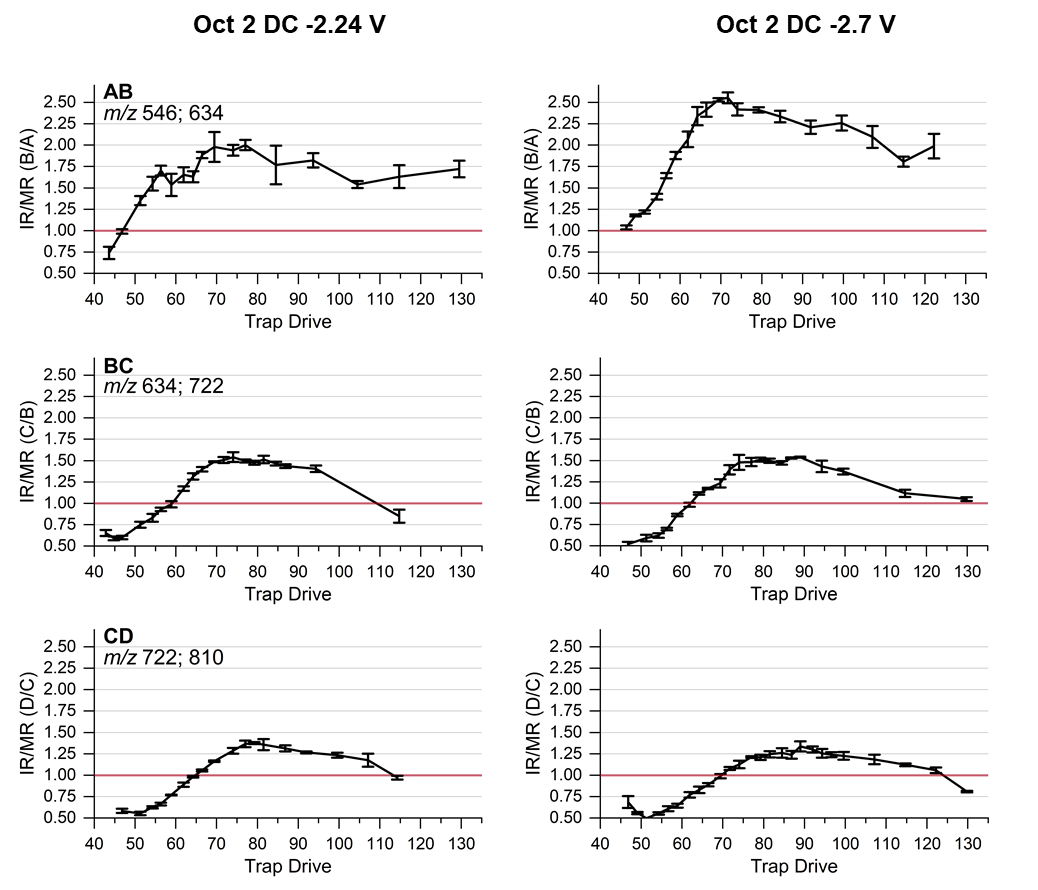


**Fig. S3** Intensity ratios,normalized for a equimolar mixture (IR/MR) of the binary mixtures AB, BC and CD, calculated from the measurement data displayed in Fig. 3 of the body text. [M-H]^-^ of *m*ABA‑labeled cellobiose deriviatives (Fig. 2 body text) are considered ions. n= 3

1. **Influence of octopole voltages on ion transportation**

Table S1 shows the measurement parameters which were chosen to analyze the influence of the Oct 2 DC as well as Oct RF on the ion transportation. Since the kinetic energy with which the ions enter the trap depends on the applied Oct 2 DC voltage, TD was varied accordingly. TD_max_ (= TD at maximum intensity) of each of the two components of the binary mixtures was calculated according to equation *(S1)* and the midpoint TD is applied.

${TD}_{max}=0.0567 \cdot(m/z)+10.52 \cdot Oct 2 DC+4.69$ *(S1)*

**Table S1** Measurement parameters applied in order to study the influence of Oct 2 DC and Oct RF on ion transportation. Variation of Oct RF from 131 to 200 Vpp was carried out at constant Oct 2 DC of 1.74 V. Oct RF was kept at 200 Vpp during variation of Oct 2 DC and TD as listed here

| **Oct 2 DC [V]** | **TD_max_ midpoint** | | |
| --- | --- | --- | --- |
|  | **AB** | **BC** | **CD** |
| -1.74 | 56.5 | 61.5 | 66.5 |
| -2.00 | 59.2 | 64.2 | 69.2 |
| -2.24 | 61.7 | 66.7 | 71.7 |
| -2.48 | 64.3 | 69.3 | 74.3 |
| -2.70 | 66.6 | 71.6 | 76.6 |
| -2.91 | 68.8 | 73.8 | 78.8 |
| -3.11 | 70.9 | 75.9 | 80.9 |
| -3.30 | 72.9 | 77.9 | 82.9 |
| -3.49 | 74.9 | 79.9 | 84.9 |
| -3.67 | 76.8 | 81.8 | 86.8 |

1. **Cluster Stability**

Beside [M-H]^-^, cluster of these target ions with HCl (🡪[M+Cl]^-^) and NaCl, corresponding to the sodium salt of ABA plus Cl^-^ (🡪[M-H+NaCl]^-^) were observed. In Table S2 - 4 the percentage of these clusters are presented for all binary mixtures AB, BC, and CD, related to the intensity of [M-H]^-^. As can be seen, the analytes form HCl and NaCl-clusters to about the same extent. However, the HCl‑clusters of the higher methoxyethylated compound were more stable against higher Cap Exit voltages. In contrast, the NaCl-clusters did not dissociate even at higher Cap Exit voltages.

**Table S2** Percentage of detected [M+Cl]^-^ and [M-H+NaCl]^-^ of the binary mixtures AB related to the intensity of [M-H]^-^ at various Cap Exit

| **% of [M-H]^-^** | | | | |
| --- | --- | --- | --- | --- |
| **Cap Exit [V]** | **[A+Cl]^-^** | **[B+Cl]^-^** | **[A-H+NaCl]^-^** | **[B-H+NaCl]^-^** |
| -90 | 44.91 | 44.78 | 9.52 | 8.90 |
| -110 | 35.72 | 31.26 | 10.16 | 7.40 |
| -130 | 19.93 | 20.93 | 7.83 | 6.23 |
| -150 | 17.24 | 15.76 | 8.41 | 6.28 |
| -170 | 10.86 | 13.89 | 6.31 | 6.02 |
| -190 | 4.40 | 9.38 | 6.20 | 5.55 |
| -210 | 0.99 | 4.72 | 5.78 | 5.66 |
| -220 | 1.22 | 3.15 | 7.73 | 6.23 |
| -230 | 0.00 | 2.07 | 6.44 | 6.22 |
| -240 | 0.00 | 2.64 | 8.51 | 6.79 |
| -250 | 0.00 | 1.67 | 9.24 | 6.82 |
| -265 | 0.00 | 1.79 | 9.95 | 6.88 |
| -280 | 0.00 | 1.58 | 12.35 | 7.47 |

**Table S3** Percentage of detected [M+Cl]^-^ and [M-H+NaCl]^-^ of the binary mixtures BC related to the intensity of [M-H]^-^ at various Cap Exit

| **% of [M-H]^-^** | | | | |
| --- | --- | --- | --- | --- |
| **Cap Exit [V]** | **[B+Cl]^-^** | **[C+Cl]^-^** | **[B-H+NaCl]^-^** | **[C-H+NaCl]^-^** |
| -90 | 19.65 | 13.92 | 7.27 | 7.03 |
| -110 | 15.77 | 11.77 | 5.32 | 6.67 |
| -130 | 13.53 | 10.22 | 5.25 | 6.36 |
| -150 | 12.94 | 9.62 | 5.62 | 6.30 |
| -170 | 12.65 | 10.26 | 5.53 | 7.08 |
| -190 | 9.52 | 8.44 | 5.78 | 6.36 |
| -210 | 4.68 | 6.84 | 5.29 | 6.40 |
| -230 | 1.59 | 3.20 | 5.58 | 6.74 |
| -250 | 0.73 | 1.25 | 6.40 | 7.50 |
| -265 | 0.73 | 0.19 | 6.21 | 7.38 |
| -280 | 0.75 | 0.00 | 6.43 | 7.35 |

**Table S4** Percentage of detected [M+Cl]^-^ and [M-H+NaCl]^-^ of the binary mixtures CD related to the intensity of [M-H]^-^ at various Cap Exit

|  | **% of [M-H]^-^** | | | |
| --- | --- | --- | --- | --- |
| **Cap Exit [V]** | **[C+Cl]^-^** | **[D+Cl]^-^** | **[C-H+NaCl]^-^** | **[D-H+NaCl]^-^** |
| -90 | 32.79 | 34.49 | 10.61 | 10.43 |
| -110 | 32.80 | 34.78 | 11.15 | 10.86 |
| -130 | 30.56 | 32.23 | 12.03 | 11.11 |
| -150 | 25.95 | 28.31 | 10.55 | 9.58 |
| -170 | 26.47 | 30.21 | 11.31 | 12.84 |
| -190 | 23.14 | 30.77 | 13.49 | 13.58 |
| -210 | 22.83 | 29.53 | 13.40 | 13.92 |
| -230 | 14.44 | 24.77 | 13.78 | 13.63 |
| -250 | 6.10 | 15.68 | 12.70 | 13.19 |
| -265 | 2.64 | 8.67 | 13.03 | 12.48 |
| -280 | 0.43 | 2.70 | 13.53 | 11.90 |

1. **Reproducibility of the IR/MR**

To prove the reproducibility of the IR/MR at the finally selected measurement parameters for the binary mixtures of AB, BC and CD (see Table 2, body text), the mixtures were measured at three different concentrations five times on three days. The *intraday* as well as the *interday* mean and standard deviation were determined (Table S5).

**Table S5** Reproducibility of the IR/MR of the binary mixtures of *m*ABA‑labeled cellobiose derivatives (AB, BC, CD, see Fig. 2, body text). IR is defined as the intensity of the higher methoxyethylated cellobiose, divided by the lower methoxyethylated one, i.e. B/A, C/B, and D/C, respectively

| **Mixture** | **Total concentration (M)** | ***Intraday* mean ± SD**  **(n= 5)** | | | ***Interday* mean ± SD**  **(n=5; p=3)** |
| --- | --- | --- | --- | --- | --- |
|  |  | **Day 1** | **Day 2** | **Day 3** |  |
| AB | 10^-5^  10^-6^ | 0.95 ± 0.02  0.99 ± 0.03 | 0.93 ± 0.02  0.95 ± 0.02 | 0.99 ± 0.01  0.97 ± 0.01 | 0.96 ± 0.03  0.97 ± 0.02 |
| BC | 10^-5^  10^-6^ | 1.07 ± 0.01  1.06 ± 0.01 | 1.09 ± 0.01  1.07 ± 0.02 | 1.05 ± 0.01  1.10 ± 0.01 | 1.07 ± 0.02  1.08 ± 0.02 |
| CD | 10^-5^  10^-6^  10^-7^ | 1.11 ± 0.01  1.14 ± 0.01  1.09 ± 0.01 | 1.11 ± 0.02  1.12 ± 0.01  1.09 ± 0.03 | 1.10 ± 0.01  1.12 ± 0.01  1.10 ± 0.02 | 1.11 ± 0.01  1.13 ± 0.01  1.09 ± 0.01 |

1. **Expanded Uncertainty of the IR/MR**

The expanded uncertainty (95.0 %) of IR/MR was calculated according to Type A evaluation. The final IR/MR were determined from all measurements (10^-6^ – 10^‑4^ M, mixture CD also 10^‑7^ M), which were recorded at the optimal measurements settings, as weighted average (equation S2). For the determination of the expanded uncertainty, both the standard deviation of the HPLC reference method and the MS measurements, i.e. the external standard deviation of the weighted average of IR/MR, were taken into account (equation S3).

$\bar{x}= \frac{\sum_{i=1}^{n} p_{i}x_{i}}{\sum_{i=1}^{n} p_{i}}$ *(S2)*

*x_i_*: average of the IR/MR of individual series of measurements

*n*: number of series of measurements; n= 8 -12

*p_i_*: reciprocal square of the standard deviation of the average values *​​x_i_;* *p_i_=* 1/*s_i_*^2^

An external standard deviation was determined for the weighted average:

$s_{ext}^{2}= \frac{1}{n-1} \frac{\sum_{i=1}^{n} p_{i} \left( x_{i}-\bar{x} \right)^{2}}{\sum_{i=1}^{n} p_{i}}$ *(S3)*

1. **TD measurements under optimized conditions and correction factors**

The TD measurements were repeated under the selected optimized conditions for Cap Exit and octopole voltages (Oct) (see Table 2, body text). If there are no discrimination effects, the compounds should have the same intensities at their individual TD_max_. Obviously, this was not the case (Fig. S4). Furthermore, the midpoint of TD_max_ is not located in a robust IR/MR range. An alternative approach could be to measure at a TD, where IR/MR does not change significantly with TD. This behavior is found at higher TD values, above TD 77 for all cellobiose derivative mixtures AB, BC, and CD, measured at the optimized Cap Exit and Oct settings. However, here the IR/MR is above 1.2 for all three mixtures. Consequently, in case of measuring under these conditions, correction factors must be applied for every component of the cellobiose ethers (∆ *m/z* 44). The factors obtained for the intensity increase with each substitution of Me by MeOEt in DP2 are presented in Table S6. For more information on the establishment of these factors, see body text (*Application of the optimized instrumental settings to methoxyethylmethyl-cellulose*).


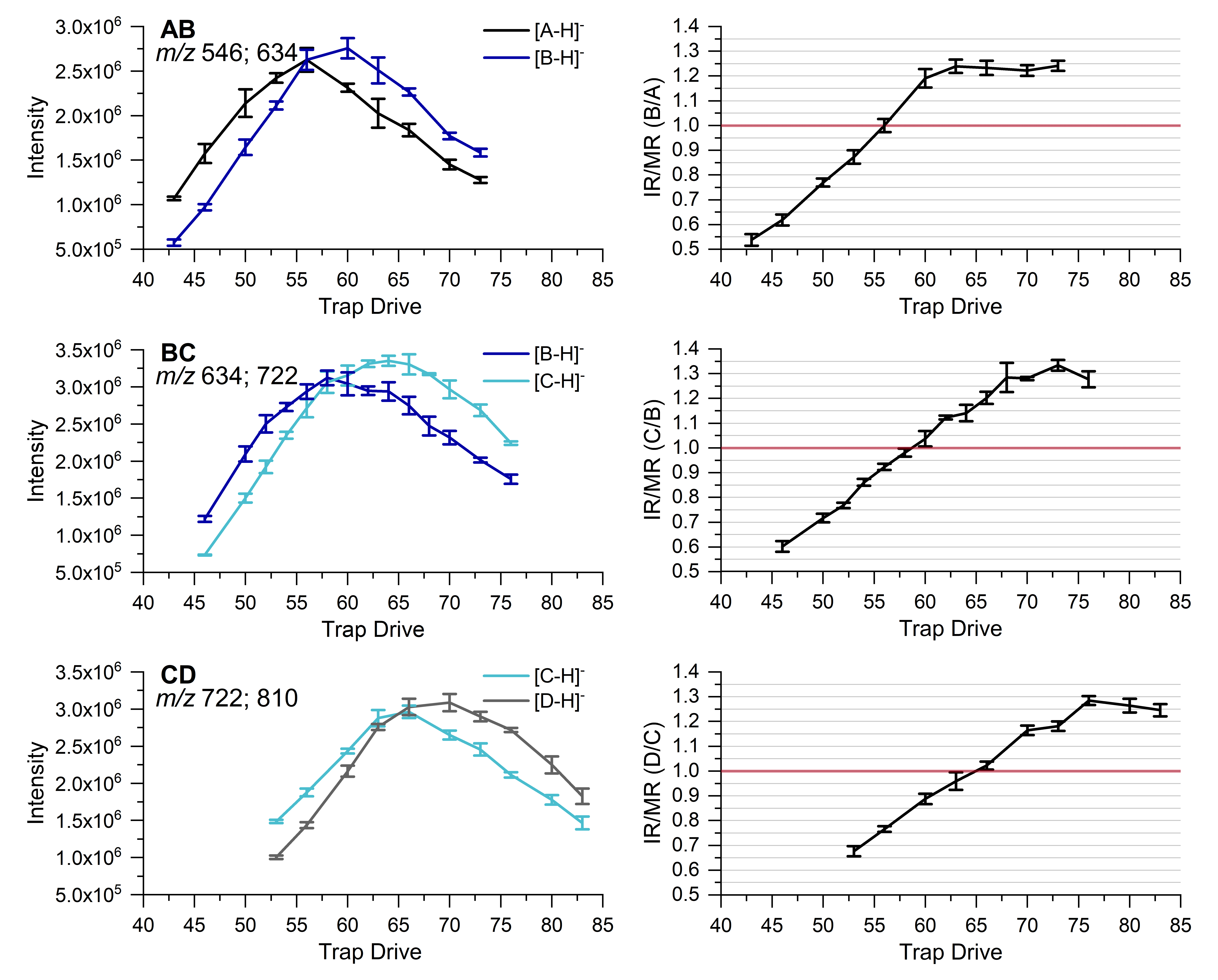


**Fig. S4** Left: Absolute intensities recorded at the optimized Cap Exit and Oct 2 settings (see Table 2, body text) for the binary mixtures of cellobiose derivatives (AB, BC, CD, see Fig. 2, body text) at a total concentration of 1 $\cdot$ 10^-6^ M in ACN/H_2_O (90/10 v/v) by ESI-IT-MS (syringe pump infusion) at various Trap Drive (RF amplitude of the ring electrode). Right: IR/MR, i.e. corrected for the exact molar ratios (MR) according to the reference data given in Table 1 (body text); n=3. Further measurement parameters see body text

**Table S6** Factors of intensity increase for the consecutive constituents of *m*ABA-labeled MeOEt/Me cellobiose, derived from HEMC, by ESI-IT-MS at TD 77 (robust area), for further measurement parameters see Table S7. The factors all refer to *m/z* 546 and were applied to correct the signal intensities prior to normalization of the substitution pattern in MeOEt/Me-cellobiose

| ***m/z*** | **Intensity increase factor referred to *m/z* 546** |
| --- | --- |
| 546 | **1.000** |
| 590 | 1.125 |
| 634 | 1.265 |
| 678 | 1.424 |
| 722 | 1.601 |
| 766 | 1.801 |
| 810 | 2.027 |

1. **Substituent Distribution of MeOEt/Me celluloses**

The MeOEt distribution of permethoxyethylated methylcellulose MC1 was analyzed under standard conditions (TM1000; Trap Drive Level 100 %; Compound Stability 1000 %; Cap Exit 280 V; Oct 2 DC 2.7 V; Oct RF 200 Vpp, TD 99.6) and under the optimized so-called expert conditions (see Table S7). The substituent patterns determined with both measurement settings are shown in Fig. S5, together with the corresponding Me-*d_3_* distribution determined for the perdeuteromethylated MC1 (reference Me-*d_3_*, [1]). The root mean square (RMS) value was calculated (equation *S4*), to express the overall deviation between the reference data (Me‑*d_3_*) and the distribution obtained under expert and standard conditions, respectively.

Furthermore, DP2 of MC1 was measured at a constant TD value (TD 77, robust range) by correction of peak areas according to the intensity increase with MeOEt, given in Table S6 (Fig. S6).

[1] Schleicher S, Lottje IR, Mischnick P. Impact of instrumental settings in electrospray ionization ion trap mass spectrometry on the analysis of multi-CH_3_-/CD_3_-isotopologs in cellulose ether analysis: a quantitative evaluation*.* Anal Bioanal Chem. 2022;414:1279–1296*.*

10.1007/s00216-021-03767-w

**Table S7** Measurement parameters for the overlapping segments in the analysis of the hydroxyethyl distribution of HE(M)C after permethylation, partial hydrolysis and *m*ABA labeling by ESI-IT-MS (syringe pump infusion, negative mode). Further parameters: Oct 2 DC -1.74 V, Oct RF 200 Vpp, Cap Exit ‑280 V except for *m/z* <730, see footnotes). For general ESI parameters see body text (I*nstrumentation*)

| **DP** | ***n*(HE)** | ***m/z*** | **TD (calculated)^a^** | **Applied TD values** | | | | | |
| --- | --- | --- | --- | --- | --- | --- | --- | --- | --- |
|  |  |  |  |  |  |  |  |  |  |
| **2** | 0 | 546 | 54.0 |  |  |  |  |  |  |
|  | 1 | 590 | 56.5 | 56.5^b)^ |  |  |  |  |  |
|  | 2 | 634 | 59.0 |  |  |  |  |  |  |
|  | 3 | 678 | 61.5 |  | 61.5^c)^ |  |  |  |  |
|  | 4 | 722 | 64.0 |  |  |  |  |  |  |
|  | 5 | 766 | 66.5 |  |  | 66.5^d)^ |  |  |  |
|  | 6 | 810 | 69.0 |  |  |  |  |  |  |
|  |  |  |  |  |  |  |  |  |  |
| **3** | 0 | 750 | 65.6 |  |  |  |  |  |  |
|  | 1 | 794 | 68.1 | 68.1 |  |  |  |  |  |
|  | 2 | 838 | 70.6 |  |  |  |  |  |  |
|  | 3 | 882 | 73.1 |  | 73.1 |  |  |  |  |
|  | 4 | 926 | 75.6 |  |  |  |  |  |  |
|  | 5 | 970 | 78.0 |  |  | 78.0 |  |  |  |
|  | 6 | 1014 | 80.5 |  |  |  |  |  |  |
|  | 7 | 1058 | 83.0 |  |  |  | 83.0 |  |  |
|  | 8 | 1102 | 85.5 |  |  |  |  | 86.8 |  |
|  | 9 | 1146 | 88.0 |  |  |  |  |  |  |
|  |  |  |  |  |  |  |  |  |  |
| **4** | 0 | 954 | 77.1 |  |  |  |  |  |  |
|  | 1 | 998 | 79.6 | 79.6 |  |  |  |  |  |
|  | 2 | 1042 | 92.1 |  |  |  |  |  |  |
|  | 3 | 1086 | 84.6 |  | 84.6 |  |  |  |  |
|  | 4 | 1130 | 87.1 |  |  |  |  |  |  |
|  | 5 | 1174 | 89.6 |  |  | 89.6 |  |  |  |
|  | 6 | 1218 | 92.1 |  |  |  |  |  |  |
|  | 7 | 1262 | 94.6 |  |  |  | 94.6 |  |  |
|  | 8 | 1306 | 97.1 |  |  |  |  |  |  |
|  | 9 | 1350 | 99.6 |  |  |  |  | 99.6 |  |
|  | 10 | 1394 | 102.1 |  |  |  |  |  |  |
|  | 11 | 1438 | 104.6 |  |  |  |  |  | 104.6 |
|  | 12 | 1482 | 107.1 |  |  |  |  |  |  |

${TD}_{max}=0.0567 \cdot(m/z)+10.52 \cdot Oct 2 DC+4.69$

1. Cap Exit -150 V
2. Cap Exit -250 V
3. Cap Exit -280 V

*(S4)*

*x_i_ (exp)*: experimental methoxyethyldistribution for the permethoxyethylated MC

*x_i_ (Me-d_3_)*: corresponding Me-*d_3_* distribution for the perdeuteromethylated MC (reference Me‑*d_3_*)


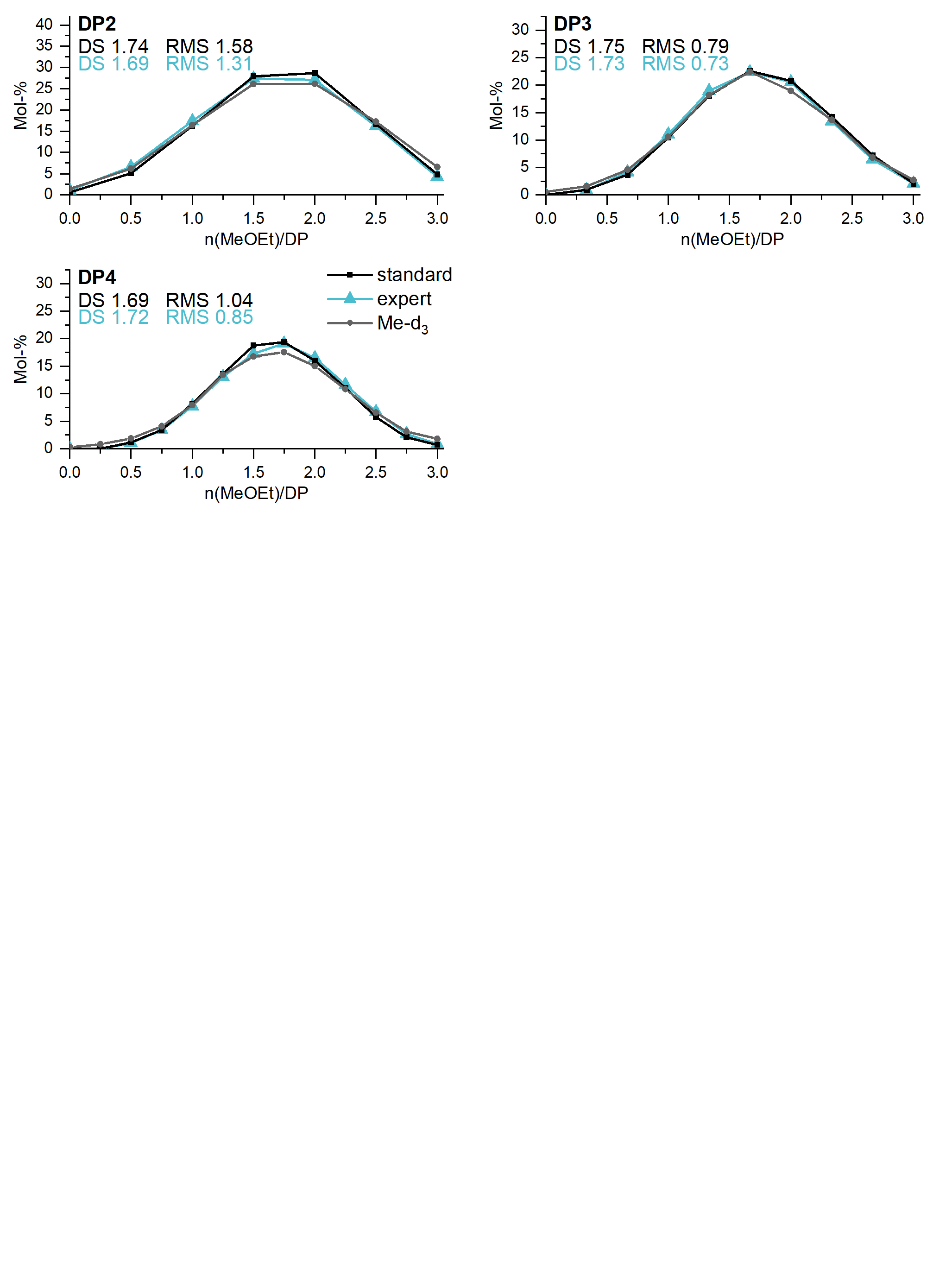


**Fig. S5** Methoxyethyl distribution of MeOEt/Me celluloses (DP2-4) obtained by ESI-IT-MS of *m*ABA‑labeled COS, derived from permethoxyethylated MC1 (DS_Me_ 1.29, DS_MeOEt_ 1.71) measured by syringe pump infusion under standard conditions (TD 99.6; Cap Exit ‑280 V; Oct 2 DC ‑2.7 V; Oct RF 200 Vpp) and expert conditions (see Table S7). The Me-*d_3_* distribution obtained after perdeuteromethylation of MC1 according to [1] is used as reference data. The deviation of the results obtained under expert and under standard conditions from these reference data is given as root mean square (RMS). n= 3


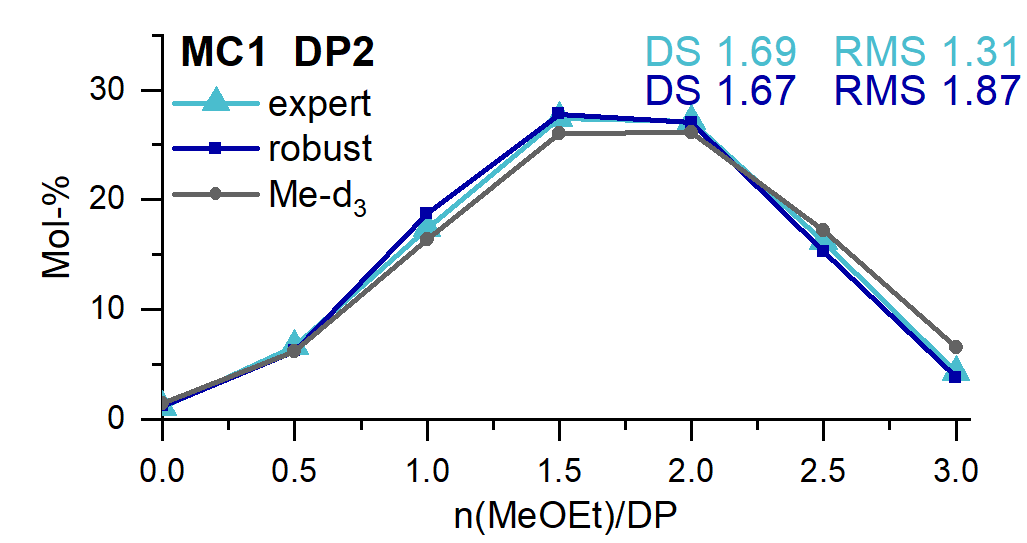


**Fig. S6** Methoxyethyl distribution of *m*ABA‑labeled cellobiose, derived from permethoxyethylated MC1 (DS_Me_ 1.29, DS_MeOEt_ 1.71) by ESI-IT-MS measured by syringe pump infusion under expert conditions (see Table S7) and at a constant TD value (TD 77, robust range) with correction of peak areas according to the intensity increase with MeOEt, given in Table S6. Further measurements parameters were analogous to expert conditions. The distribution of MC1 obtained after perdeuteromethylation (Me-*d_3_*) according to [1] is used as reference data. The deviation of the results obtained under expert and under robust conditions from these reference data is given as root mean square (RMS). n= 3
